# Mapping the microstructure of human cerebral cortex in vivo with diffusion MRI

**DOI:** 10.1038/s42003-025-08523-9

**Published:** 2025-07-22

**Authors:** Amir Sadikov, Hannah L. Choi, Jaclyn Xiao, Lanya T. Cai, Pratik Mukherjee

**Affiliations:** 1 Radiology and Biomedical Imaging, University of California, San Francisco, CA USA; 2 Graduate Group in Bioengineering, University of California, San Francisco, CA USA

**Keywords:** Brain, Diffusion tensor imaging

## Abstract

Despite advances in diffusion MRI, which have led to remarkable progress in mapping white matter of the living human brain, our understanding of cerebral cortical microstructure in vivo and its relationship to macrostructure, myeloarchitecture, cytoarchitecture, chemoarchitecture, metabolism, and function lag far behind. We present neuromaps of 21 microstructural metrics derived from diffusion tensor, diffusion kurtosis, mean apparent propagator, and neurite orientation dispersion and density imaging of the young adult cerebral cortex. These 21 metrics are explained by four composite factors that correspond to diffusion kurtosis (intracellular volume fraction/neurite density), isotropic diffusion (free water fraction), heterogenous diffusion (extracellular volume fraction) and diffusion anisotropy (neurite orientation dispersion). We demonstrate how cortical microstructure follows cytoarchitectural and laminar differentiation, aligns with the macroscale sensory-fugal and sensorimotor-association axes, and contributes to functional brain networks, neural oscillatory dynamics, neurotransmitter receptor/transporter distributions, and cognition and behavior.

## Abstract (web summary)

Cortical maps of diffusion MRI microstructure follow cytoarchitectural and laminar differentiation, align with sensory-fugal and sensorimotor-association axes, and reflect oscillatory dynamics, neurotransmitter distributions, and cognition/behavior.

## Introduction

Most of our knowledge of mammalian brain microstructure and connectivity has traditionally derived from microscopic analysis of animal models and ex vivo human brain specimens. This historical state of affairs led Crick and Jones to call for the development of technologies to map the microarchitecture and connectivity of the in vivo human brain in their article “The Backwardness of Human Neuroanatomy”^1^. This perspective proved to be timely, since the following year saw the publication of the rank-2 tensor representation of diffusion magnetic resonance imaging (dMRI) by Basser et al.^2^. Over the past three decades, diffusion tensor imaging (DTI), and higher-dimensional representations such as the rank-4 tensor (diffusion kurtosis imaging: DKI) and the mean apparent propagator (MAP-MRI) have enabled in vivo mapping of white matter microstructure and connectivity in the human brain with increasing power^3–6^. However, despite the remarkable progress in understanding white matter, “backwardness” remains in characterizing in vivo human cerebral cortex using dMRI due to limitations of signal-to-noise ratio (SNR), spatial and angular resolution, and microscale sensitivity. Hence, in vivo exploration of the human neocortex has been largely limited to macroscale measurements of volume, thickness, curvature, and surface area as well as a few cytoarchitectural or myeloarchitectural features, including iron content from susceptibility mapping^7^ and myelin content from T1/T2 mapping^8^.

The evolution of MRI hardware and software advances pioneered by the Human Connectome Project (HCP)^9^ have broken the SNR barrier with millimeter spatial resolution and higher angular resolution at multiple diffusion-weighting strengths that interrogate the tissue architecture at smaller spatial scales than conventional DTI used for white matter mapping. This opened in vivo human cortical imaging to the full panoply of microstructural metrics available from low- and high-dimensional representations such as DTI, DKI, and MAP-MRI, and biophysical models such as neurite orientation dispersion and density imaging (NODDI)^10^. An initial study has outlined the regional variation of DTI and NODDI microstructural metrics across the human cerebral cortex and its relationship with measures of macrostructure (cortical thickness), myelination (T1/T2 ratio) and cytoarchitecture (von Economo structural types)^11^. Recent clinical research also suggests advantages of cortical dMRI over traditional macrostructural measures such as thickness in disorders such as Alzheimer's disease^12–14^.

Despite this early progress, to our knowledge, there has not yet been a comprehensive investigation of how dMRI-derived cortical microstructural mapping relates to molecular, cellular, metabolic, electrophysiological, and functional variation across the human cerebral cortex. Recent breakthroughs have been achieved in generating multimodal maps of the cortex across spatial scales by integrating gene expression data and transcriptomics from the Allen Brain Atlas, neurotransmitter receptor and transporter densities as well as metabolic information from positron emission tomography (PET), electrophysiological data from magnetoencephalography (MEG), hemodynamic function from blood oxygenation level-dependent (BOLD) functional MRI (fMRI), myelination from T1/T2 ratio MRI, and macrostructure from MRI volumetrics^9,15–21^. However, conspicuously missing from these new “neuromaps” is the diversity of microstructural information available from state-of-the-art dMRI.

The aim of this work is to distill the multitude of metrics from common signal representations (DTI, DKI, MAP-MRI) and tissue models (NODDI) widely used in dMRI literature into a lesser number of explanatory factors and investigate their organization across the cortex, thereby integrating cortical microstructure into the neuromaps framework. We leverage the latest advances in dMRI preprocessing, including machine learning-based denoising, motion and image artifact correction, and outlier replacement, to achieve cortical microstructural test-retest reliability comparable to traditional macrostructural metrics such as cortical thickness. We combine high-resolution DTI, DKI, MAP-MRI, and NODDI data with multimodal neuromaps to show how regional cortical microstructure corresponds to molecular features such as neurotransmitter receptor and transporter densities, mesoscale features such as Mesulam’s hierarchy of laminar differentiation^22^, the macroscale sensorimotor-association (SA) axis of evolutionary and childhood cortical development^23^, electrophysiological oscillatory dynamics across the full spectrum of frequency bands, as well as cognition and behavior across many domains. We also demonstrate that the 21 different dMRI metrics investigated can be distilled into four explanatory factors that correspond to intracellular volume fraction, extracellular volume fraction, free water fraction and neurite orientation dispersion, replicated in two independent dMRI datasets and generalized across different ranges of diffusion-weighting strengths. These findings are a step forward in resolving the “backwardness” of human neuroanatomy.

## Results

**Fig. 1:**
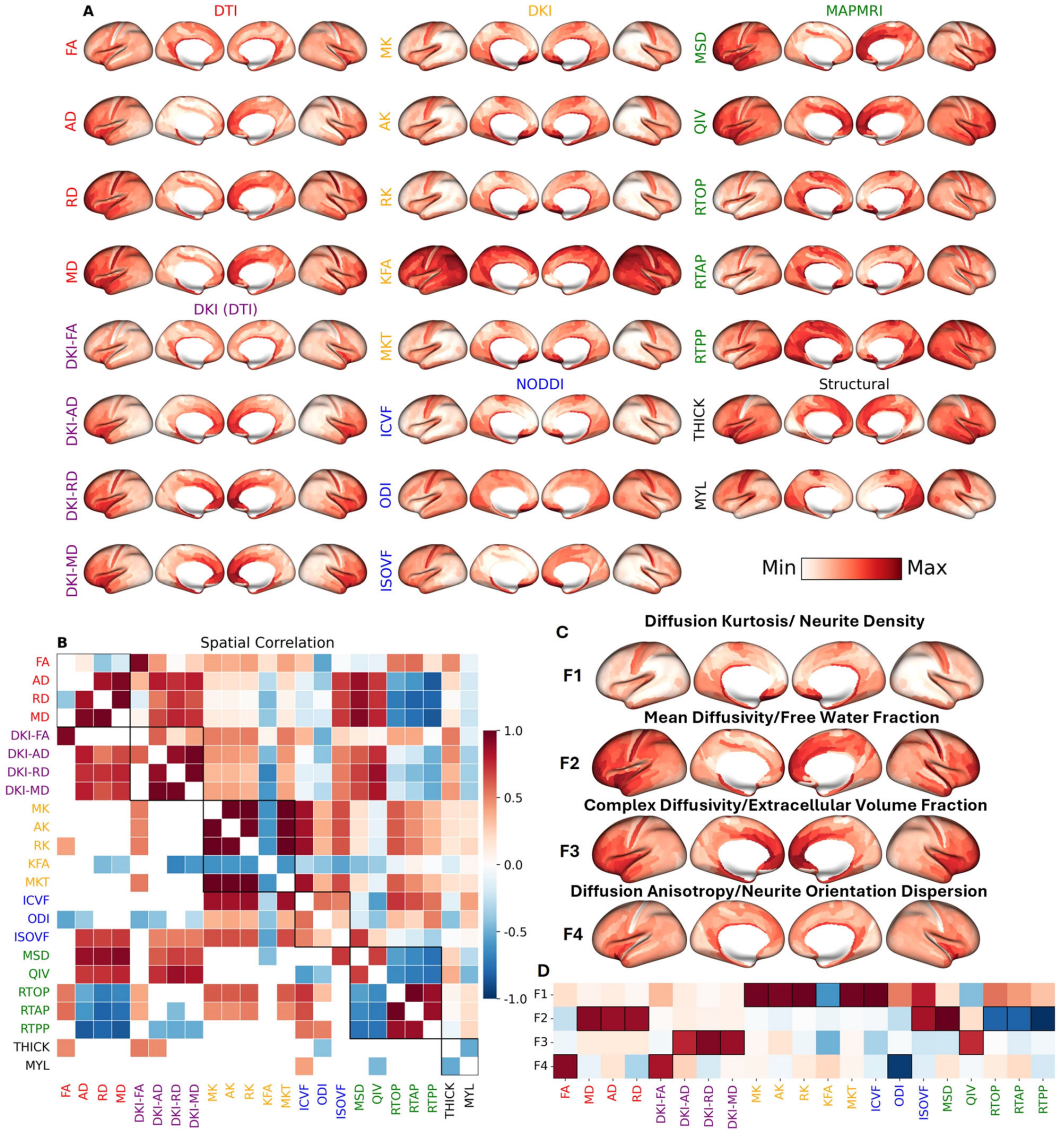
The group-averaged cortical microstructural profile of the HCP-YA dataset. **A** 21 group-averaged microstructural maps with (**B**) Pearson correlation (lower triangular: significant correlations), **C** factors & **D** loadings. We found four factors: F1-diffusion kurtosis/neurite density (MK, AK, RK, KFA, MKT, ICVF), F2-isotropic diffusivity/free water fraction (AD, MD, RD, ISOVF, MSD, RTOP, RTAP, RTPP), F3-complex diffusivity/extracellular volume fraction (DKI-AD/MD/RD, QIV) & F4-anisotropy/neurite orientation dispersion (FA, DKI-FA, ODI). Red = positive correlation/loading; Blue = negative correlation/loading.

To investigate cortical microstructure, we first took a group-averaged profile of the Human Connectome Project - Young Adult (HCP-YA) dataset, parcellated into 360 Glasser regions: FA, AD, RD, and MD from DTI; DKI-FA, DKI-AD, DKI-RD, DKI-MD, MK, AK, RK, MKT, KFA from DKI; ICVF, ODI, and ISOVF from NODDI, MSD, QIV, RTOP, RTAP, and RTPP from MAP-MRI; cortical thickness (THICK) and myelin (MYL) (Fig. 1A, refer to Supplementary Table S1 for abbreviations). The group comprises 962 healthy young adults (age range: 20–35, 55% female) after quality control exclusions. In addition to the group-average, we also measured the intersubject variability (Supplementary Fig. S1) and the laterality index (Supplementary Fig. S2) for each of the metrics. The most strikingly asymmetric anatomic feature of cortical gray matter is the right lateralization of the diffusivities (AD, MD, RD) from DTI and MSD from MAP-MRI in the medial prefrontal cortex, with corresponding left lateralization of FA from DTI, KFA from DKI and the return probabilities from MAP-MRI.

### Interrelatedness of microstructural metrics

To investigate the relationship between the microstructural metrics, region-wise Pearson correlation coefficients were computed between each metric (Fig. 1B). Our dimensionality reduction analysis revealed that the 21 dMRI microstructural metrics could be explained by four underlying factors (Fig. 1C). The first factor (F1) is diffusion kurtosis (MK, AK, RK, KFA, MKT), which positively correlated with the intracellular volume fraction ICVF, also known as neurite density, and explains 32.8% of the variance. The second factor (F2) is isotropic diffusivity (ISOVF), which represents free water fraction and is positively correlated with all the DTI diffusivities (AD, MD, RD) and the mean-squared distance (MSD) from MAP-MRI, but negatively correlated with the return probabilities from MAP-MRI (RTAP, RTOP, RTPP) and accounts for 28.5% of the variance. The third factor (F3) is complex diffusivity (QIV), which captures the heterogeneous microenvironments of the extracellular volume fraction and is therefore positively correlated with the DKI diffusivities (DKI-AD/MD/RD), explaining 15.2% of the variance. The fourth factor (F4) is diffusion anisotropy (FA, DKI-FA) which is negatively correlated with the neurite orientation dispersion index ODI from NODDI and accounts for 12.8% of the variance. In total, these four factors explained close to 90% of the total variance. We confirm their robustness to alternative dimensionality reduction techniques, such as independent component analysis (ICA), which yielded similar independent components (ICs) that were significantly correlated with their respective factors (Supplementary Fig. S3). IC1-IC4 explained 26.1%, 35.7%, 40.6%, and 21.0% of the variance, respectively, which sum to more than 100% since ICA does not enforce orthogonality. The laterality maps confirm that F2 (isotropic diffusion) is most right lateralized in the medial prefrontal cortex whereas F4 (anisotropic diffusion) is most left lateralized in the same region, as might be inferred from the individual dMRI metrics that contribute to these composite factors (Supplementary Fig. S2). There is also right lateralization of F1 (kurtotic diffusion) from the underlying DKI (AK, MK, RK, MKT) and NODDI (ICVF) metrics in medial prefrontal cortex that, although not quite as pronounced as F2, corresponds even more closely in anatomical extent to the left lateralization of F4.

### Microstructure diverges along the sensorimotor-association axis

**Fig. 2:**
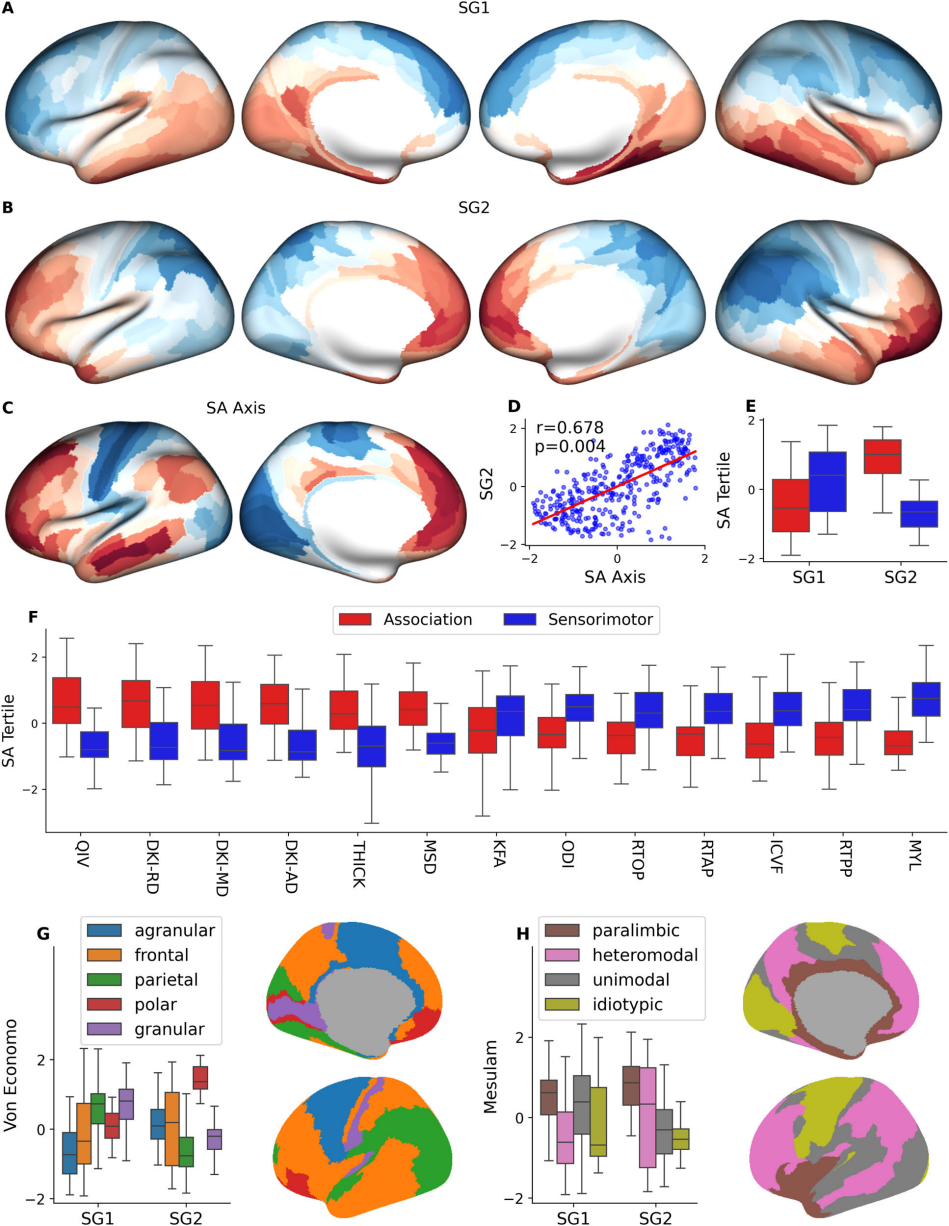
Cortical microstructure diverges along the sensorimotor-association axis and reflects cytoarchitectural classes & laminar differentiation. **A** The first and **B** second structural gradients (SG1 & SG2) derived from the dMRI measures (red and blue indicate opposing ends) along with myelin and cortical thickness. **C** The Sensorimotor-Association (SA) axis (blue indicates sensory regions, red indicates association areas) and **D** the correlation between SG2 and SA axis is statistically significant under spin permutation testing (*r *= 0.687, *p *= 0.004). The top tertile (60 regions with the highest rank) and bottom tertile (60 regions with the lowest rank) are taken to be the association tertile and sensorimotor tertile respectively. **E** Both SG1 (*t *= 4.4, *p *= 2.9e−05) and SG2 (*t* = 11.4, *p *= 2e−20) had statistically significant divergence between sensorimotor and association regions. **F** Cortical metrics significantly diverged between association and sensorimotor regions: QIV (*t *= 10.2, *p *= 5.4e−18), DKI-RD (*t *= 8.0, *p *= 5.1e−12), DKI-MD (*t *= 7.9, *p *= 5.1e−12), DKI-AD (*t *= 7.5, *p *= 3.2e−11), THICK (*t *= 7.3, *p *= 1.3e-10), MSD (*t *= 9.1, *p *= 1.7e−14), KFA (*t *= −2.7, *p *= 0.01), ODI (*t *= 4.3, *p *= 5.8e−05), RTOP (*t *= −4.8, *p *= 7.9e−06), RTAP (*t *= −5.5, *p *= 3.5e−07), ICVF (*t *= 5.8, *p *= 9.8e−08), RTPP (*t *= −6.3, *p *= 1.1e−08), MYL (*t *= 10.5, *p *= 2.8e−17). Sensorimotor regions are shown in blue and association regions are shown in red. **G** SG1 (F = 19.4, *p *= 2.0e−14) and SG2 (F = 26.5, *p *= 6.2e−19) were stratified by the Von Economo cell types. **H** SG1 (F = 21.9, *p *= 4.7e−13), SG2 (F = 27.6, *p *= 9.3e−16) were also stratified by the Mesulam’s hierarchy of laminar differentiation.

The SA axis describes a principal gradient of functional brain organization, ranging from idiotypic primary sensory and motor areas at one end to transmodal association cortices involved in abstract cognition at the other^23^. The SA axis explains much of the microstructural cortical variation and provides a link to cellular, functional, and genetic markers (Fig. 2).

To capture how diffusion microstructure varies across the cortex, we apply structural gradient analysis, which characterizes the principal axes or gradients of variation within a lower dimensional embedding space. We found neurite density (ICVF), diffusion kurtoses (AK, MK, RK, MKT), and return probabilities (RTOP/RTAP/RTPP) to capture most of the variation of the first structural gradient (SG1), while the DKI-diffusivities (DKI-AD/MD/RD) as well as their higher-order counterpart QIV from MAP-MRI captured much of the variation of the second structural gradient (SG2) (Supplementary Fig. S4). Both the first two structural gradients (SG1) and (SG2) were stratified across the SA axis and SG2 had a statistically significant correlation with the SA axis $(r=0.68,{p}=\,0.004)$ as well as the maps used to derive the SA axis: areal scaling $(r=0.46,{p}=0.012)$, the principal component of gene expression $(r=-0.73,{p}=0.002)$, and the principal gradient of functional connectivity $(r=0.50,{p}=0.023)$. Individual measures of cortical microstructure and macrostructure also showed strong associations with the SA axis (Supplementary Fig. S5) and its components (Supplementary Fig. S6). The SA axis was significantly correlated with MSD (*r *= 0.462, *p *= 0.006), QIV (*r *= 0.617, *p *= 0.002), thickness (*r *= 0.564, *p *= 0.024), and myelin (*r *= −0.70, *p *= 0.003); areal scaling was correlated with DKI-FA $(r=0.66,{p}=0.001)$, DKI-AD $(r=0.48,{p}=0.007)$, ODI $(r=-0.50,{p} < 0.001),$ thickness $(r=0.79,{p} < 0.001)$, and myelin $(r=-0.46,{p} < 0.001)$; functional intersubject variability was correlated with ICVF $(r=-0.51,{p}=0.019)$, RTOP $(r=-0.41,{p}=0.029)$, RTAP $(r=-0.37,{p}=0.029)$, and RTPP $(r=-0.34,{p}=0.042)$; the principal gradient of gene expression was correlated with DKI-AD $(r=-0.74,{p}=0.016)$, DKI-RD $(r=-0.67,{p}=0.016)$, DKI-MD $(r=-0.73,{p}=0.016)$, MSD $(r=-0.46,{p}=0.022)$, QIV $(r=-0.65,{p}=0.016)$, thickness $(r=-0.69,{p}=0.008)$, and myelin $(r=0.74,{p} < 0.001)$; the principal gradient of functional connectivity was correlated with myelin $(r=-0.48,{p}=0.03)$. In addition, we found the kurtosis factor (F1) was correlated with functional intersubject variability (*r *= −0.45, *p *= 0.026); isotropic diffusion factor (F2) was correlated with the SA axis (*r *= 0.39, *p *= 0.042); complex diffusivity factor (F3) was correlated with the SA axis (*r *= 0.58, *p *= 0.042) and the principal component of gene expression (*r *= −0.72, *p *= 0.01); and anisotropy factor (F4) was correlated with areal scaling (*r *= 0.62, *p *= 0.008).

We confirm the known greater thickness and lower myelination of association versus sensorimotor cortex^23^. We further show higher DKI diffusivities (AD, MD & RD) as well as their higher-order counterpart QIV from MAP-MRI for association cortex compared to sensorimotor cortex, which implies higher complex diffusivity factor F3. Also, from MAP-MRI, the higher MSD and lower return probabilities (RTAP, RTOP & RTPP) of association versus sensorimotor cortex imply higher isotropic diffusion factor F2 as well. Association cortex also exhibited lower cellularity (ICVF) and orientation dispersion (ODI) (Supplementary Fig. S5).

All measures of microstructural intersubject CoV that showed statistically significant divergence across the SA axis (KFA, ICVF, ODI, and myelin), had lower intersubject variability in sensorimotor areas, reflecting its older phylogeny than the association areas^24^.

### Laminar differentiation and cytoarchitecture stratify cortical microstructure

Cortical microstructure was stratified by both cytoarchitecture and laminar differentiation, with SG1 and SG2 showing statistically significant differences across the von Economo structural types and the Mesulam laminar hierarchies (Fig. 2). Both Mesulam’s hierarchy of laminar differentiation and the von Economo and Koskinas structural types can be succinctly summarized in atlas form. In the von Economo atlas, granular and agranular cortex were at opposite extremes of SG1, whereas polar cortex was distinguished from parietal and granular cortex by SG2. For the Mesulam atlas, SG2 best differentiated paralimbic and idiotypic cortices. Furthermore, SG1 was correlated with Thionin staining $(r=0.38,{p}=0.015)$, specific for DNA and Nissl substance, while SG2 was correlated with Bielschowsky staining $(r=0.39,{p}=0.03)$, a silver staining method used to demonstrate fibrous elements such as neurofibrils.

**Fig. 3:**
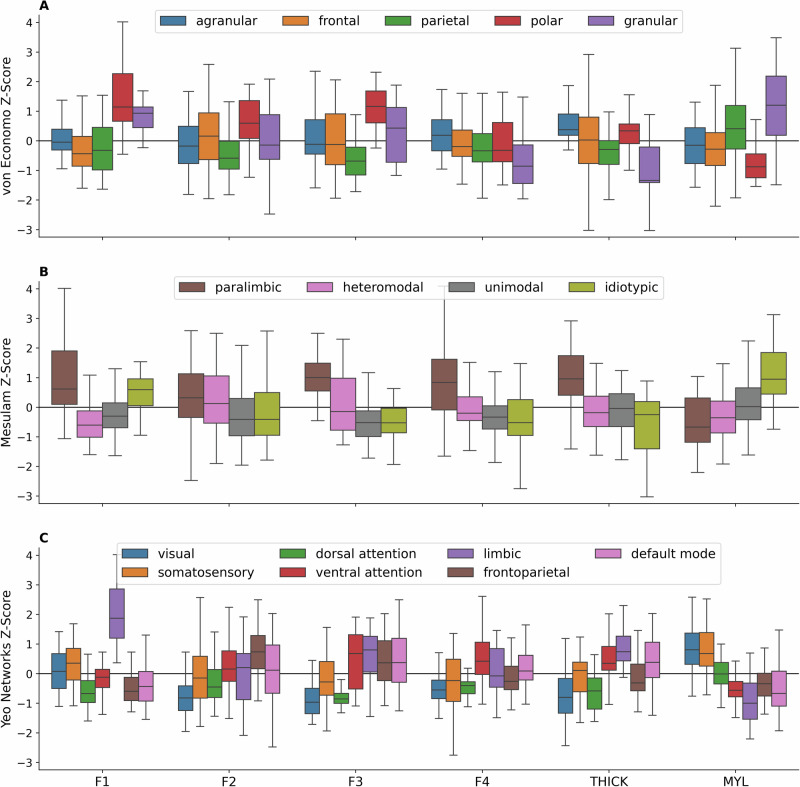
Four underlying cortical microstructural factors stratify cytoarchitectural classes, laminar differentiation and functional networks. Four dMRI factors, thickness, & myelin for (**A**) von Economo structural types, **B** Mesulam’s hierarchies & **C** Yeo fMRI networks. Polar cortex had high kurtosis (F1). Granular cortex had higher F1 but lower anisotropy (F4) than agranular cortex. Paralimbic cortex had high complex diffusivity (F3) & F4. There was more F1, but less isotropic diffusivity (F2), F3 & F4 from heteromodal to idiotypic regions. Sensory & dorsal attention networks diverged from the four association networks for F2, F3 & F4.

In addition to the structural gradients, each specific microstructural metric as well as the majority of intersubject CoVs and LIs showed statistically significant differences across both the von Economo structural types and Mesulam organizational hierarchies (Supplementary Figs. S7, S8). Much of the divergence occurred along the sensory-fugal axis, which describes the hierarchical stream of sensory information: starting in primary idiotypic areas, projecting into unimodal association areas, then heteromodal association areas, with a further stage in paralimbic areas. Paralimbic cortex was distinguished by higher complex diffusivities (F3), anisotropies (F4), kurtoses (F1), and free water (F2) but lower KFA (Fig. 3, Supplementary Fig. S8). DKI and NODDI measures were best at differentiating the other three levels of Mesulam’s hierarchy, with progressively lower F1 (kurtoses: AK, MK & RK; and cellularity: ICVF) from idiotypic to unimodal to heteromodal cortex. Conversely, F4 (anisotropy driven primarily by decreasing ODI) increased along the sensory-fugal axis and reached a peak in paralimbic cortex due to its high FA from both DTI and DKI. This aligns with the well-known decrease of cortical myelination and increase of cortical thickness along the sensory-fugal axis, a gradient that we also observed^22^.

Much like Mesulam’s paralimbic cortex, the von Economo polar regions were also distinguished by high complex diffusivities (F3), high kurtoses (F1), high free water (F2) and strikingly low KFA, but not high thickness (Fig. 3, Supplementary Fig. S7). Polar cortex was also more easily identified by high MSD and QIV, the two MAP-MRI metrics most associated with high rates of diffusivity in homogenous (F2) and heterogenous (F3) environments, respectively. Compared to agranular cortex, granular cortex was characterized by higher F1 (kurtoses: AK, MK & RK and cellularity: ICVF) and lower F4 (anisotropy due to lower FA and higher ODI). The lower cortical thickness and higher myelination of granular versus agranular cortex that we observed has been previously reported^25,26^. The higher ICVF in granular cortex agrees with previous findings in human and animal studies that reported higher neuron density in granular regions^27,28^. Synapse density, quantified via synaptic vesicle glycoprotein 2A (SV2A) binding density, was negatively correlated with ICVF $(r=-0.29,{p}=0.013)$ and myelin $(r=-0.30,{p}=0.008)$, confirming the inverse relationship between neuron density and synapse-to-neuron ratio^29^. Finally, we performed Tukey’s Honest Significant Differences (HSD) test to determine which of the pairwise differences across Mesulam’s hierarchy of laminar differentiation and the von Economo and Koskinas structural types were significant (Supplementary Tables S3, S4).

To determine the extent to which these changes in the microstructural factors are transmitted through cortical thickness or myelination, we performed mediation analysis, where we set one of the factors as the outcome, an indicator for one of the areas (e.g. idiotypic) as the input, and either cortical thickness or myelination as the potential mediator and recorded the proportion of the relationship that was mediated (Supplementary Table S5). We confirm that for most areas there is mild to moderate mediation by cortical thickness and myelination. We find high levels of mediation (over 50%) only for idiotypic cortex, specifically myelin for kurtoses (F1), mean diffusivity (F2), and complex diffusivities (F3) but thickness for anisotropy (F4). In an equivalent analysis for the von-Economo and Koskinas structural types, we found high levels of mediation (over 50%) only for thickness and anisotropy (F4) in frontal cortex and for myelin and kurtoses (F1) in granular cortex as well as complex diffusivities (F3) in agranular cortex. We also found negative values in several cases, indicating competitive mediation or suppression.

Intersubject CoV of dMRI-based metrics were lowest in idiotypic and granular regions, consistent with previous measures of intersubject variability, such as functional intersubject variability ^30^, and supporting recent theories of developmental and evolutionary expansion^23,24,30,31^. While there has been increasing evidence for lower inter-subject variability in evolutionarily older regions^32–35^, there is a need for research showing more direct links. Kurtoses (AK, MK & RK) were left-lateralized in the parietal cortex and right-lateralized in the polar and agranular cortices, possibly owing to the divergent hemispheric lateralization of visual and language processes^36,37^.

### Microstructure exhibits a diversity of organizing principles

Microstructural metrics varied based on resting state fMRI networks (Fig. 3C, Supplementary Fig. S9). In a fashion similar to both Mesulam’s hierarchy and the von Economo structural types, the Yeo fMRI networks can be distinctly summarized by a cortical atlas. We applied Tukey’s HSD test to highlight significant pairwise differences (Supplementary Table S6). The Yeo fMRI networks fell into two classes that mirrored the dichotomy of the SA axis (Fig. 1 & Supplementary Fig. S5). Visual, somatosensory and dorsal attention networks shared characteristics of sensorimotor cortex, whereas ventral attention, frontoparietal, limbic and default mode networks shared characteristics of association cortex. Specifically, the former three networks had lower isotropic (F2), complex (F3) and anisotropic (F4) diffusion than the latter four networks. This dichotomy was supported by cortical thickness, which was lower in the former three than the latter four networks, as well as by myelination, which had the reverse relationship. The dissociation between dorsal and ventral attention networks was also supported by dMRI factors F2, F3, & F4 as well as by cortical thickness, whereas, for myelination, the dorsal and ventral attention networks formed more of a continuum between morphological characteristics of sensorimotor networks versus association networks.

The limbic network showed more extreme kurtosis (F1) than other fMRI networks due to its large component of paralimbic cortex, consistent with higher neurite density or, equivalently, intracellular volume fraction. All microstructural values with their composite factors, intersubject CoVs and most LIs showed statistically significant divergence across Yeo functional networks under one-way ANOVA tests with FDR correction (Supplementary Fig. S9).

We computed adjusted coefficients of determination, expressed as a percentage, to measure the amount of variation the following organizations could explain for each microstructural metric: SA axis, Mesulam’s hierarchy, Yeo functional networks, and the von Economo structural types (Supplementary Fig. S10). We found metrics to exhibit disparate organizational hierarchies. For instance, the SA axis was unable to capture the variation of the DKI higher-order metrics, whereas the other organizing principles were. Similarly, the von Economo-Koskinas structural types were unable to explain regional variation of the MAP-MRI return probabilities (RTAP, RTOP & RTPP) but the other organizations did. DTI and DKI FA variation was captured by only Mesulam’s hierarchies and the Yeo networks; KFA was also expressed by both as well as the von Economo structural types. The diffusivities from DTI and DKI, ICVF and ODI from NODDI, MSD from MAP-MRI, and myelin and cortical thickness showcased universal organization across all parcellations. Importantly, the DKI diffusivities were more coherently organized across all hierarchies than the DTI diffusivities. Finally, we found most microstructural metrics were best explained by the Yeo functional networks and/or Mesulam’s hierarchy.

### Microstructure reflects dynamic neuronal oscillatory activity

**Fig. 4:**
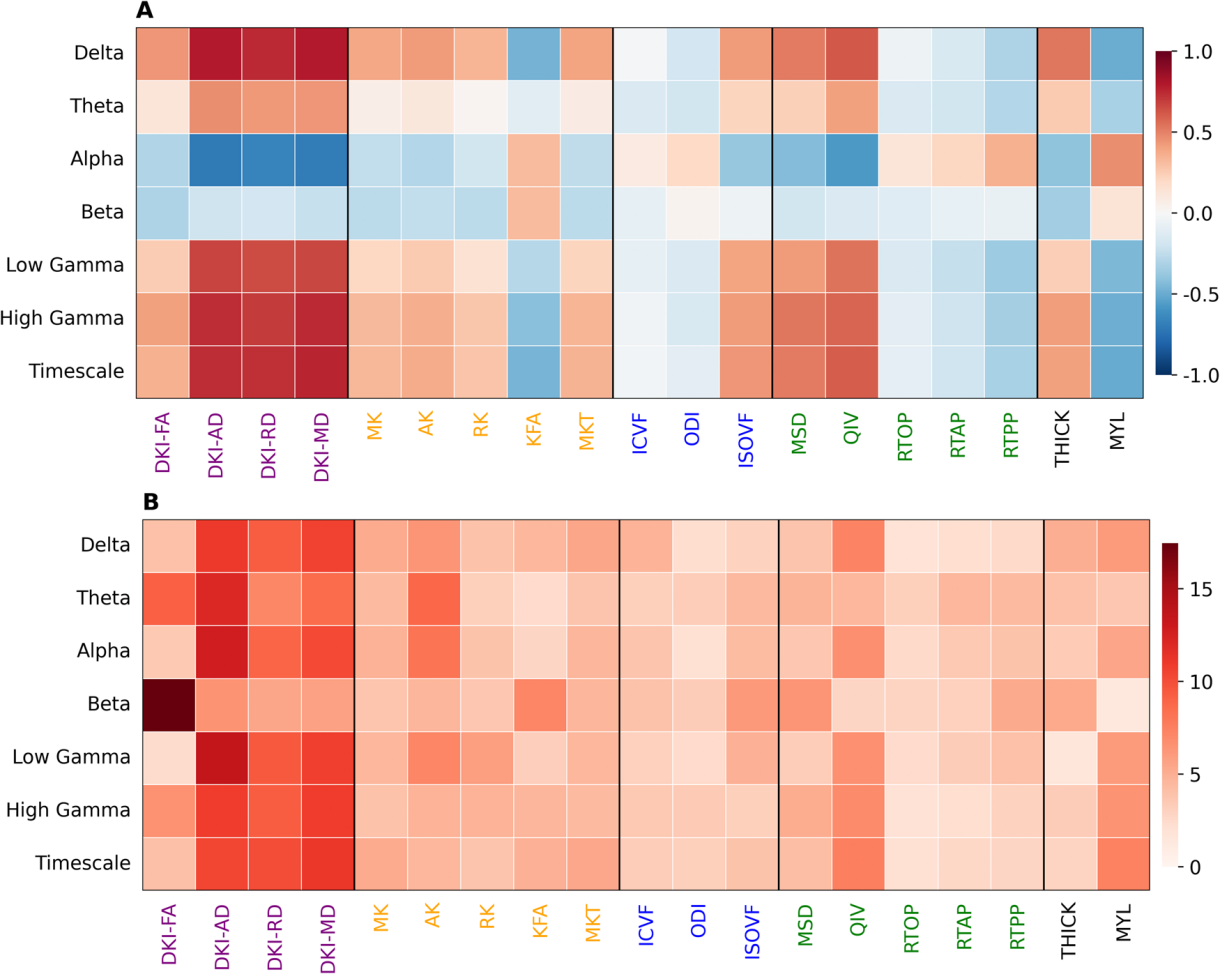
Cortical microstructure molds neural oscillatory dynamics captured by MEG. **A** Pearson Correlation coefficients between MEG power distributions for the delta (2–4 Hz), theta (5–7 Hz), alpha (8–12 Hz), beta (15–29 Hz), low gamma (30–59 Hz) and high gamma (60–90 Hz) frequency bands and intrinsic timescale and structural metrics. **B** Dominance analysis shows the relative contribution of each variable to a linear model. The DKI diffusivities and DKI-FA, specifically for the beta frequency band, played the largest roles in MEG Power and timescale prediction. Each prediction was statistically significant: delta $(r=0.92,{p}=0.0002)$, theta $(r=0.81,{p}=0.003)$, alpha $(r=0.90,{p}=0.0003)$, beta $(r=0.79,{p}=0.007)$, low gamma $(r=0.87,{p}=0.0002)$, high gamma $(r=0.88,{p}=0.0002)$.

Given that microstructural profiles exhibited hierarchical organization in the brain as well as both structural and functional connectivity, we next sought to identify whether they also reflect neuronal oscillatory dynamics measured by MEG (Fig. 4). Predictions for MEG power from every frequency band and timescale using cortical microstructure were statistically significant under FDR-corrected one-sided spin permutation testing $(0.79\, < {r} < \,0.92,{p} < \,0.007)$. Distance-dependent cross-validation analysis confirmed the robustness of our model (Supplementary Fig. S11). Divergence across the SA axis explained 28–47% of the variance of the prediction at different spectral types, except for the beta frequency band where it only explained 4%. Dominance analysis showed the predictions were mostly mediated by the DKI diffusivities, except for the beta frequency band, where DKI-FA played the strongest role.

### Microstructure captures neurotransmitter receptor and transporter densities, cognition and behavior

Along with neuronal dynamics, we found microstructure to also capture some of the distribution of neurotransmitter receptors and transporters (Supplementary Fig. S12, refer to Supplementary Table S2 for abbreviations). DKI measures were significantly correlated with the concentration of 5-HTT, D1, DAT, NMDA, and VAChT. Cortical thickness was significantly correlated with 5HT1a, 5HT4, 5HT6, CB1, D1, D2, H3, M1, mGluR5, and MOR while myelin was significantly correlated with 5HT4, CB1, M1, and MOR. Dominance analysis showed cortical thickness playing the largest role in multivariate prediction for receptor densities. Multivariate predictions of receptor or transporter density using microstructure were statistically significant for every receptor/transporter under FDR-corrected one-sided spin permutation testing $(0.64\, < {r} < \,0.85,{p} < \,0.002)$. Cross-validation test predictions confirmed the robustness of our models (Supplementary Fig. S13). We found stratification across Mesulam’s hierarchy of laminar differentiation to account for 2–50% of the variance of our predictions across receptors & transporters, with better explanatory power for transporters (5HTT, DAT, NET & VAChT) than their corresponding neurotransmitter receptors.

**Fig. 5:**
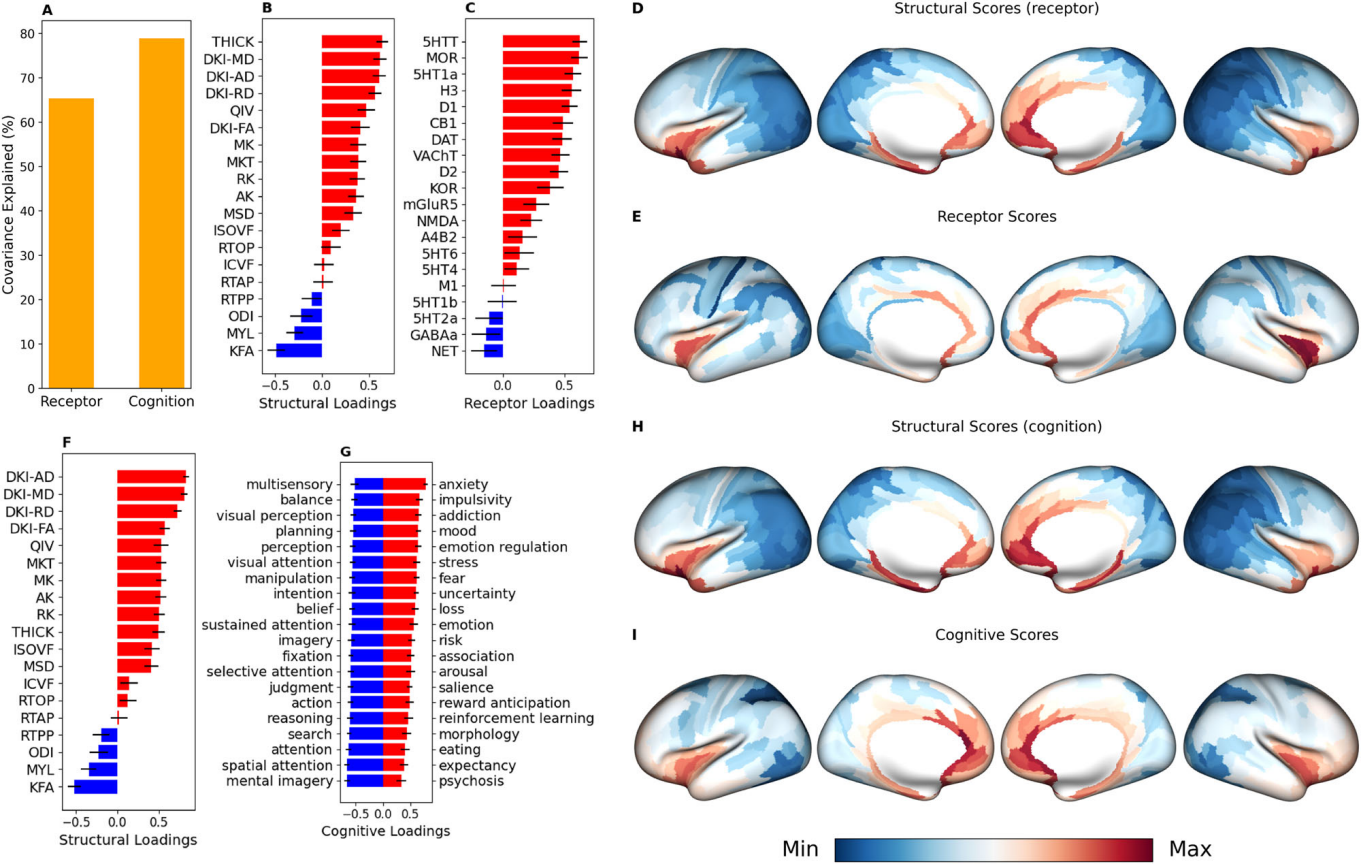
Microstructure reflects neurotransmitter receptor & transporter densities and cognitive functioning. **A** PLS correlation between receptor/transporter densities and microstructural metrics revealed a statistically significant principal gradient (*p *= 0.002) that explained 65.4% of the covariance. Equivalent PLS correlation analysis between cognitive task activation maps and microstructural metrics revealed a principal gradient (*p *= 0.001) that explained 78.9% of the covariance. **B** Structural and **C** receptor/transporter loadings showed universally positive weightings, except for KFA, myelin, ODI and GABAa and NET. **D** Structural and **E** receptor/transporter scores had strong correspondence with each other (*r *= 0.678, *p *= 0.0002) and showed the most positive weighting in paralimbic cortex and the most negative weighting in idiotypic and unimodal cortex. **F** Structural loadings from PLS analysis with cognitive maps mirrored those from PLS analysis with receptor/transporter densities and the two corresponding structural scores (**D**, **H**) were almost identical (*r *= 0.995, *p *< 0.0001). **G** The twenty most positive cognitive loadings were associated with emotional and reward processing, while the twenty most negative loadings were associated with sensory processing/integration, attention, judgement, and planning. **I** The cognitive scores were well correlated with the structural scores (*r *= 0.83, *p *= 0.0004) and even the receptor/transporter scores (*r *= 0.62, 0.0004). Both the receptor $(r=-0.62,{p}=0.0036)$ and structural $(r=-0.76,{p}=0.0012)$ scores (**D**, **E**) showed statistically significant correspondence with the first principal component of gene expression. Similarly, the cognitive $(r=-0.69,{p}=0.006)$ and the structural $(r=-0.72,{p}=0.01)$ scores (**H**, **I**) also showed statistically significant association with the first principal component of gene expression.

Partial least squares (PLS) correlation analysis revealed that a statistically significant principal gradient $(p=0.002)$ explained 65.4% of the covariance between neurotransmitter receptor/transporter densities and cortical microstructure (Fig. 5). The structural and receptor/transporter scores had high spatial correspondence with each other $(r=0.679,{p}=0.0002)$, revealing a divergence across the sensory-fugal axis with strong positive weighting in paralimbic regions and strong negative weightings in idiotypic and unimodal cortex. Both the receptor/transporter $(r=-0.62,{p}=0.0036)$ and structural $(r=-0.76,{p}=0.0012)$ values showed statistically significant correspondence with the first principal component of gene expression. Microstructural and receptor/transporter loadings showed uniformly positive weightings, except for KFA, myelin, and ODI as well as NET and GABAa, suggesting a mostly convergent relationship.

Structural loadings from PLS analysis with sensory, cognitive and behavioral maps mirrored those from PLS analysis with receptor/transporter densities and the two corresponding structural scores were almost identical (Fig. 5). The twenty most positive cognitive loadings were associated with emotional and reward processing, while the twenty most negative loadings were associated with sensory processing/integration, attention, judgement, and planning. The cognitive scores were well correlated with the structural scores (*r *= 0.83, *p *= 0.0004) and even the receptor/transporter scores (*r *= 0.62, 0.0004). Similar to the receptor/transporter and corresponding structural values, the cognitive $(r=-0.69,{p}=0.006)$ and the structural $(r=-0.72,{p}=0.01)$ scores also showed statistically significant association with the first principal component of gene expression.

### Consistency of cortical microstructure metrics from diffusion MRI

We used the test-retest portion of HCP-YA (*n* = 38) to assess the consistency of our microstructural metrics, which we quantify via test-retest CoV and ICC over the Glasser parcellation (Supplementary Figs. S14, S15). Most of the microstructural metrics derived from the high quality, multi-shell dMRI acquisitions of the HCP-YA dataset have consistency equivalent to widely used macrostructural measures, such as cortical thickness. Every metric, except ISOVF and myelin, had a maximum regional CoV of 4%. Myelin had CoV above 4% only in the left and right posterior orbitofrontal cortical (OFC) complex. Other than ISOVF, which had a mean test-retest CoV of 9.4%, every other metric had a mean test-retest CoV less than 2%. We observed the lowest mean CoV in RTPP and the DKI diffusivities (AD, RD & MD), with CoV below 1%. All microstructural metrics maintained a regional ICC above 0.7, other than DKI-AD, MK, AK, RK, and MKT in fewer than four out of 360 regions each. All metrics had a mean ICC above 0.84, with DKI-FA, FA, ICVF, ODI, thickness, and myelin having mean ICCs above 0.9.

### Replication and generalization to the MGH-USC dataset

For replication purposes in an independent dataset acquired on a different model of MR scanner with a different dMRI acquisition protocol, we computed the same 21 cortical microstructural metrics from the MGH-USC data using all four shells at b = 1000, 3000, 5000 & 10000 s/mm^2^ (except for the first four DTI maps at b = 1000 s/mm^2^ only) and also using only the b = 1000 & 3000 s/mm^2^ shells to match the same shells of HCP-YA (Supplementary Fig. S16). The four explanatory factors in the HCP-YA dataset were replicated in both the full MGH-USC data as well as the limited MGH-USC data using equivalent explanatory factor analysis (Supplementary Fig. S17). The correlation between the HCP-YA and MGH-USC data is greater than 0.90 for 19 of 21 cortical dMRI metrics for both the full and limited MGH-USC data, with only KFA and RTOP falling slightly below that level for the full MGH-USC data that included diffusion-weighting strengths much greater than in the HCP-YA data (Supplementary Fig. S17). Scatterplots showed especially good correspondence between the two datasets for DTI metrics, which were limited to the b = 1000 shell s/mm^2^, and for the NODDI metrics ICVF and ISOVF even when using all four MGH-USC shells (Supplementary Fig. S18). For other dMRI metrics, there was a systematic bias towards lower diffusivity measurements when using the full MGH-USC data as well as towards lower MSD and higher return probabilities from MAP-MRI. These effects can all be explained by greater signal rectification at the noise floor for the two very high b-value shells of the full MGH-USC acquisition that are not acquired in the HCP-YA dataset^38,39^.

## Discussion

Starting from the work of Brodmann^40^, von Economo^26^, and others^41–43^, considerable effort has gone into cataloging the cytoarchitectural, myeloarchitectural, and laminar structure of the human brain. Recently, advanced imaging methods have enabled investigations into connectomics, where the cortex is modeled as a graph of homogeneous nodes with functional and/or structural edges. However, this simplification abstracts away the microstructural variation intrinsic to the mammalian brain. This research is an effort to add and integrate dMRI-derived microstructural information into a rich compendium of the cortex that includes molecular, cellular, laminar, dynamic, and functional attributes^15^. Assembling and combining the multimodal properties of the cortex will be essential to investigate how the complex microstructure and connectivity of the brain give rise to emergent properties and lead to cognition and complex behaviors.

The ability of dMRI parameters to differentiate cortical areas stems from their sensitivity to the local microenvironments experienced by water molecules. Variations in cell density, the extracellular space, neurite orientation dispersion, and other factors will differentially hinder and restrict water diffusion, leading to distinct dMRI-based signatures. Recent dMRI research in human brain white matter in vivo has found three underlying factors that correspond well to our F1, F2 & F4 factors in gray matter^44^. Since this prior work included DTI, DKI & NODDI, but not MAP-MRI, the sensitivity to heterogenous diffusivity factor F3 that is anchored by the QIV metric may be limited. Differences in the tissue microenvironment of gray matter versus white matter might be another explanation that is not mutually exclusive.

We found prominent links between in vivo dMRI cortical metrics and cytoarchitecture and laminar differentiation. While previous literature found high-resolution ex vivo maps of DTI and MAP-MRI parameters to reveal laminar substructures^45^ and show correlation with histological markers of cytoarchitecture^46^, we now show that high-resolution in vivo human dMRI exhibits differences among Mesulam’s hierarchy of laminar differentiation and among von Economo structural types. Importantly, many of these differences were not strongly mediated by cortical thickness or myelination, suggesting that cortical thickness and myelin, in most cases, only explain a small part of the relationship between the cortical microstructural factors from dMRI and Mesulam’s hierarchy of laminar differentiation as well as the von Economo and Koskinas structural types. In addition to the well-known increase of cortical thickness and decrease of myelination along the sensory-fugal axis, we additionally show decreasing neurite density (F1) and fiber orientation dispersion (F4) but increasing free water fraction (F2). This helps explain the relatively large mediation effects of myelination on F1 in idiotypic cortex (76%) among the Mesulam classes and on F1 in granular cortex (60%) among the von Economo types, since these are the most myelinated regions of neocortex due to their high neurite density. The histologically-based BigBrain cortical atlas illustrates that increasing thickness along the sensory-fugal axis is due to expanding layers III, V and, to a lesser extent, VI^47^. Our microstructural findings agree with microscopy studies of these laminae in macaque brain demonstrating increasing axonal field size, dendritic arborization and number of synapses resulting in more neuropil along the sensory-fugal axis^48–51^. Cortical dMRI microstructure showed clear organization across the SA axis and several of its components. For instance, we found complex diffusivity factor (F3) to be significantly correlated with the principal component of gene expression, agreeing with previous literature showing gene expression to reflect distributions of various cell types across regions^19^. We also found kurtoses factor (F1), which corresponds to neurite density, to be significantly negatively correlated with functional intersubject variability, likely because neurite density was higher in the sensory regions and lower in the association areas. Similarly, we found anisotropy factor (F4) and areal scaling to be significantly positively correlated, likely because anisotropy is lowest in the myelinated sensorimotor regions and highest in association cortices, agreeing with known theories of areal expansion^52^.

Interestingly, diffusivities derived from multi-shell dMRI acquisition (DKI-AD, DKI-MD & DKI-RD) had greater differences than their single-shell DTI equivalents between sensorimotor and association regions and among Mesulam’s hierarchy, von Economo structural types, and Yeo functional networks (Supplementary Figs. S7–S9). This observation differs from the prior diffusion MRI of white matter literature in which signal rectification effects at the higher diffusion-weighting factors due to strongly anisotropic tissue can distort diffusivity measurements^38^ and suggests an advantage of applying high-b factor dMRI to cerebral cortex to better detect signal from smaller spatial scales, in agreement with recent work^53,54^.

Cortical patterns of microstructure clearly divided the seven Yeo fMRI networks into a sensory group (somatosensory, visual and dorsal attention) and an association group (ventral attention, default mode, frontoparietal and limbic). This classification was not as clear with cortical thickness and was nonexistent with cortical myelination. These categorizations fit with the known functional roles of these networks, except for the two attention networks that are closely related to both sensory and higher-order cognitive functions^55^. The pronounced microstructural differences between the dorsal and ventral attention networks suggest the former is closer to the sensory pole of the sensory-fugal axis whereas the latter is closer to the opposite pole of transmodal/heteromodal information processing. This result is concordant with a recent investigation demonstrating that maturation of the ventral attention network in childhood is crucial for attainment of adult cognitive and behavioral profiles^56^. Further research is needed to more definitively establish the functional significance of this microstructural dichotomy between the dorsal and ventral attention networks.

The dMRI-derived microstructural metrics were more predictive of MEG than conventional measures such as cortical thickness and myelination. Dominance analysis showed the diffusivities (AD, MD, & RD) derived from DKI to be the most explanatory metrics across all MEG frequency bands and the intrinsic timescale, except for the beta band for which FA was dominant. The SA axis explained up to 47% of the variation of the multivariate prediction of MEG power and intrinsic timescale, except at the beta frequency band. Delta and gamma band power arise more strongly from association cortex, especially in the frontal lobes, than sensorimotor regions^57^. This leads to a strong positive correlation with the diffusivities. The reverse is true for the alpha band, which arises primarily from sensorimotor areas of occipital and parietal lobes; therefore, the correlation with the diffusivities is strongly negative. Theta band oscillations, which arise largely from the temporal lobes including the hippocampus, are intermediate, with a weaker positive correlation with the diffusivities than delta or gamma bands. The overall negative correlation of beta band oscillatory power with the diffusivities is consistent with its sources from sensorimotor planning regions; however, beta band activity has no significant correlation with the SA axis. It is notable that high gamma band activity has a stronger positive correlation with the diffusivities than low gamma band activity. This presumably mirrors the known greater bias of high gamma band activity towards the frontal lobes compared to the low gamma band. This also helps explain the strongly positive correlation of the diffusivities with the MEG intrinsic timescale. These findings are consistent with recent work showing that the temporal hierarchy of intrinsic timescales in MEG converges with the spatial hierarchy along the SA axis, with longer timescales in “core” association regions and shorter timescales in “peripheral” sensorimotor regions^58^. A full mechanistic explanation for how cortical microstructure affects MEG oscillatory activity is out of the scope of this report and would require dedicated neurophysiological research.

Mesulam’s hierarchy of laminar differentiation explained up to 50% of the variation in some neurotransmitter receptor densities. In addition, PLS correlation analysis suggests that the sensory-fugal axis reflects the relationships between microstructure, cognition, and neurotransmitter receptor/transporter distributions. Microstructural metrics with the highest divergence across the sensory-fugal axis, such as the DKI diffusivities and myelin, had the greatest loading magnitudes. The density of neurotransmitter receptors/transporters closely linked with mood regulation were highest in fugal areas, including 5-HTT, 5-HT1a, cholinergic, dopaminergic, cannabinoid, and opioid receptors. In addition, affective processes were positively weighted while attentive processes were negatively weighted in paralimbic and insular regions. Finally, structural, cognitive, and receptor/transmitter scores were all closely aligned with the sensory-fugal axis and significantly correlated with the principal component of gene expression, hinting at the importance of gene expression architecture behind these relationships.

While T1w/T2w ratio is a widely used, accessible in vivo proxy for myelin content^8^ and is sufficient for our analysis, which relies on the relative distribution of myelin across the cortex, other techniques, such as quantitative magnetization transfer, quantitative susceptibility mapping, synthetic MRI, ultra-short echo-time imaging, and PET among others may be considered in future work^59^. Due to the difficulty in modeling gray matter microstructure, we mostly used signal representations such as DTI, DKI, and MAP-MRI, which do not make many underlying assumptions. The only tissue model we did make use of is NODDI, which we recognize is ill-posed to fully capture gray matter microstructure due to the short time scale of diffusion across cell membranes. However, we believe that because NODDI continues to be widely used across dMRI literature, particularly in the clinical context, its inclusion is merited. Furthermore, models more specific to the underlying biology of gray matter, such as NEXI^53,54^, require acquisitions with multiple diffusion times and ideally at a greater number of b-values with greater diffusion weighting. High-dimensional q-space representations such as MAP-MRI could also benefit from greater diffusion weighting and sampling, but current clinical applications are limited to acquisitions similar to the HCP-YA^60^. This is currently impractical for any large patient cohort for which microstructural measures can be derived. While NODDI is suboptimal for gray matter, we found that its metrics of cellularity, fiber orientation dispersion and free water content were still often correlated with structural and functional properties of human cerebral cortex and accounted for three of the four explanatory factors from the joint analysis of microstructural metrics that also included DTI, DKI and MAP-MRI. This was replicated in two independent dMRI datasets and also generalized to different ranges of diffusion-weighting strengths in the limited versus full MGH-USC acquisition. The dominance analysis we utilized to gauge the relative importance of the various microstructural metrics is limited to linear relationships, as is the PLS correlation analysis for cognitive and neurotransmitter receptor/transporter associations. Future work in larger and more diverse datasets is needed to investigate nonlinear interactions across subjects. Our analyses focused on group-averaged microstructural profiles and did not investigate potential effects of demographic variables such as sex or age. Given known sex and age differences in brain structure and function, exploring how these influence cortical microstructure and its lateralization patterns is an important avenue for future research.

To minimize partial volume effects, we replaced voxels with dMRI metric values greater than one standard deviation from the cortical gray matter ribbon using nearest-neighbor sampling. While this approach enhances the specificity of our findings to the gray matter compartment, it may also discard voxels that reflect true microstructural variance. Future investigations with higher resolution dMRI could confirm our findings and provide insights into the complex microstructural architecture of the gray matter-white matter interface^61^.

As dMRI progresses to ultra-high fields (at and above 7 Tesla) with higher performance gradients and radiofrequency systems, greater spatial resolutions will allow for laminar-specific examination of neural microstructure^62^, while greater q-space resolutions will allow for fitting to more complex and more faithful models of the gray matter. Integrating mesoscale T1 and T2 relaxometry with dMRI in a multidimensional framework might also offer improved microstructural visualization of cortical laminae, as recently demonstrated for ex vivo human neocortex^63^. Advances in generative artificial intelligence show promise in leveraging high-quality dMRI data collected on specialized MRI hardware for improved imaging speed, quality and reproducibility on lower performance clinical MRI scanners for both white matter and gray matter^64^. This should enable high-resolution cortical dMRI in larger patient cohorts for improved diagnosis, prognosis and treatment monitoring.

## Methods

All code and data used to perform these analyses can be found at https://github.com/ucsfncl/diffusion_neuromaps. Volumetric images are included in the neuromaps package^15^.

### Microstructural data acquisition

We used structural and diffusion preprocessed data from the S1200 release of the Human Connectome Project Young Adult (HCP-YA) dataset^9^ to create cortical microstructural profiles. We exclude any subjects with quality control issues due to anatomical anomalies, segmentation and surface errors, temporal head coil instability, and model fitting irregularities^65^. As a result, our analysis comprises 962 subjects, 38 of whom also have retest data.

First, the diffusion data was denoised via Marchenko-Pastur Principal Component Analysis (MPPCA) denoising^66^ followed by Rician debiasing^67^. We fit the diffusion tensor imaging (DTI)^2^ model with only the b = 1000 s/mm^2^ shell and diffusion kurtosis imaging (DKI)^5^, Neurite Orientation Dispersion and Density Imaging (NODDI)^10^, and Mean Apparent Propagator-MRI (MAP-MRI)^6^ models with the full dMRI acquisition. The DTI, DKI, and MAP-MRI fitting was performed via dipy^68^; the NODDI fitting was performed via AMICO^69^. We fit the DKI model with ordinary least-squares fitting using the Splitting Conic Solver from CVXPY^70^; the MAP-MRI model using a radial order of 6, Laplacian regularization of 0.05, and a positivity constraint; and NODDI fitting using a modified parallel diffusivity value of 1.1 × 10^−3^ mm^2^/s to better capture gray matter microstructure^71^. In total, we collected the following metrics: fractional anisotropy (FA), axial diffusivity (AD), radial diffusivity (RD), mean diffusivity (MD) from DTI; DKI-FA, DKI-MD, DKI-RD, DKI-AD, mean kurtosis (MK), radial kurtosis (RK), axial kurtosis (AK), mean kurtosis tensor (MKT), and kurtosis fractional anisotropy (KFA) from DKI; neurite density index (i.e., intracellular volume fraction: ICVF), orientation dispersion index (ODI), and isotropic volume fraction (ISOVF) from NODDI; q-space inverse variance (QIV), mean-squared displacement (MSD), return-to-origin probability (RTOP), return-to-axis probability (RTAP), and return-to-plane probability (RTPP) from MAP-MRI; cortical thickness measured from FreeSurfer recon-all^72^ and myelin as expressed in T1w/T2w MRI.

We used Nilearn’s vol_to_surf functionality to sample our volumetric microstructural maps uniformly between MSMAll pial and white matter fsLR-32k surfaces^20,73^, while masking out any voxels not in the cortical ribbon. For MAP-MRI derived values, we conducted additional outlier detection by excluding any voxels greater than 4.5 median absolute deviations from the median due to the presence of some extreme outliers. For all microstructural values, we excluded any cortical voxels greater than one standard deviation away from the mean to ensure that the analysis focused on voxels with values representative of the typical cortical ribbon, removing less typical or partial volume affected voxels at the edges of the distribution within the cortex^11,74^. We filled in any missing values via nearest neighbor sampling from known values on the MSMAll midthickness surface^75–77^. Finally, we parcellated the structural maps using the Glasser atlas^20^ to reduce noise and improve interpretability with further subdivisions via the Mesulam^22^ and von-Economo-Koskinas atlases^25,26^ to investigate their cortical organization.

To assess repeatability, we computed the test-retest coefficient of variation (CoV) and the two-way mixed, single measures, absolute agreement intraclass correlation (ICC)^78^. The test-retest CoV was computed as the subject-average standard deviation over sessions divided by the average value taken over the subjects and sessions. For the ICC, the variation between measurements was found using the test-retest portion and the variation between subjects was found using the whole dataset. To assess inter-subject variability, we computed the inter-subject CoV, taking the measurement error found in the test-retest dataset into account^30^. Finally, the laterality index was computed as $\frac{{x}_{{LH}}-{x}_{{RH}}}{{x}_{{LH}}+{x}_{{RH}}}$, with positive values indicating left lateralization and negative values indicating right lateralization.

We found the structural covariance networks (SCNs)^79–82^ for each metric by computing the correlation between pairs of gray matter regions across all subjects. We also computed an SCN that would encompass all metrics by z-scoring the microstructural data and then computing the correlation between pairs of gray matter regions across all subjects and metrics. We derived our structural gradients via degree-normalized Laplacian embedding^13,83,84^. We focused our analysis on the first two gradients, disregarding the first steady-state eigenvector.

We performed dimensionality reduction on the 21 dMRI metrics via factor analysis to retain interpretability. We chose a minimum residual approach with promax rotation, keeping all factors that explained more than 1% of the variation. The four resulting factors (F1–F4) were named by assigning the metrics that had the greatest loadings. We confirmed robustness to alternative dimensionality reduction via independent component analysis (ICA), implemented using FastICA (scikit-learn) with four components. We rearranged the independent components (ICs) and flipped their signs to correspond to the four factors.

### MEG maps

Magnetoencephalography (MEG) power across six frequency bands: delta (2–4 Hz), theta (5–7 Hz), alpha (8–12 Hz), beta (15–29 Hz), low gamma (30–59 Hz), high gamma (60–90 Hz) and intrinsic timescale were collected as part of the HCP-YA project^9^ and sourced from the neuromaps library^15^. Previous publications have detailed the data collection and processing^18,85^. The maps were parcellated into Glasser regions.

### PET maps

Positron emission tomography (PET) maps were collected from multiple studies^86–125^ and sourced from the neuromaps library^15^. For further information, see the following publication^17^. In all cases, only healthy controls were used. The maps are proportional to receptor/transporter density, and following convention, we refer to their values as receptor/transporter densities. Previous work has verified the consistency of the receptor/transporter densities via comparison with autoradiography data^17^. The maps were parcellated into Glasser regions.

### Mediation analysis

Mediation analysis was performed to quantify how much of the relationship between the factors and Mesulam’s hierarchy of laminar differentiation and the von Economo and Koskinas structural types was mediated by either cortical thickness or myelin. We set one of the factors as the outcome variable, an indicator function for a specific region (e.g. 1 for idiotypic cortex and 0 otherwise) as the input, and either cortical thickness or myelin as the mediator. We recorded the proportion mediated (%) as the proportion of the relationship between the presence or absence of a particular region (e.g. idiotypic) and any one of the factors that is transmitted through or mediated by either cortical thickness or myelin.

### Dominance analysis

Dominance analysis was performed to determine the relative contribution of each variable to a multiple linear regression model^126^. This was conducted for each input variable by measuring the average increase in the coefficient of determination when adding the single input variable across all possible combinations of input variable. The total dominance was expressed as a percentage of the adjusted coefficient of determination of the complete model. The robustness of the multiple linear regression model was assessed distance-dependent cross-validation, where the closest 75% of regions was taken to be the training set and the further 25% of regions was the test set^17^. By meticulously calculating and averaging each variable’s unique contribution to model fit across all possible combinations of other predictors, dominance analysis can quantify the predictability of each variable.

### Permutation testing

We used spin permutations, which preserves spatial autocorrelation, to generate null distributions for testing statistical significance^127^. We extracted Glasser and DK region centroid coordinates from the spherical projection of the fsLR-32k surface and then applied a random rotation and, for bihemispheric statistical testing, applied reflection before and after the rotation to the right hemisphere. Original parcels were reassigned to their closest rotated parcels for each permutation. All multiple hypothesis tests also underwent false discovery rate (FDR) correction. Unless otherwise stated, our significance level was set to 5%.

### Cognitive brain maps

fMRI task-activation maps^128^ were obtained using NeuroQuery^129^, a meta-analytic machine learning tool that predicts the spatial distributions of neurological observations given an input string. We selected 123 cognitive terms from the Cognitive Atlas^130^, previously used in the neuroscience literature^17,85^.

### Partial least squares correlation analysis

Partial least squares (PLS) correlation analysis^131,132^ was used to relate microstructure to neurotransmitter receptor densities and functional activations via the pyls package. PLS projects two datasets onto orthogonal sets of latent variables with maximum covariance. The scores were computed by projecting the original data onto their respective weights and the loadings are the Pearson’s correlation coefficient between the values and the scores. The significance of the latent variable was assessed via spin permutation testing. We restricted our analysis to only the first latent variable.

### MGH-USC dataset

We used the structural MRI and preprocessed dMRI data from the MGH-USC dataset to establish reproducibility. The MGH-USC dataset consists of 35 healthy adults (20–59 years old) with structural (T1w, T2w) and diffusion data acquired on a 3T CONNECTOM scanner, capable of producing gradients up to 300 mT/m. The dMRI acquisition consisted of four shells: 1000 s/mm^2^ (64 directions), 3000 s/mm^2^ (64 directions), 5000 s/mm^2^ (128 directions), and 10,000 s/mm^2^ (256 directions) at 1.5 mm isotropic resolution. dMRI was corrected for gradient non-linearity, head motion, and eddy current artifacts. Structural MRI was also corrected for gradient non-linearity. Extensive information on the scanner, acquisition protocols, and preprocessing performed can be found in Fan et al.^133^

FreeSurfer recon-all^72^ was used to generate native pial and white matter surfaces, which were subsequently resampled to the fsLR-32k mesh. dMRI metric volumes and T2w MRI were registered to T1w MRI via boundary-based registration^134^. dMRI metrics were subsequently computed using the same methodology as for the HCP-YA dataset. Cortical thickness was resampled using the metric resample functionality in the connectome workbench. Myelin contrast was derived from the T1w/T2w ratio and volume-to-surface mapping was conducted using connectome workbench functionality^8^.

We fit our microstructural metrics on a limited subset of the dMRI acquisition (only b = 1000 s/mm^2^ and b = 3000 s/mm^2^) to emulate the b-value range of the HCP-YA dataset and the full acquisition to investigate how the dMRI metrics change with the addition of high b-value shells. We applied explanatory factor analysis to the MGH-USC Dataset, using the same methods as in the HCP-YA analysis.

### Statistics and reproducibility

Our analysis consists of 962 subjects after exclusion due to quality control issues, 38 of whom had retest data. We established the consistency and reliability of dMRI microstructural metrics via ICC and CoV measurements using test-retest data. Furthermore, we were able to produce similar group-averaged dMRI cortical maps and factors using the MGH-USC dataset, with and without the inclusion of high b-value shells (b = 5000 s/mm^2^, b = 10000 s/mm^2^). We confirmed the robustness of our factor analysis by performing ICA and obtaining similar components. For all correlation analysis, we used spin permutations to generate the null distributions. We performed one-way ANOVA to determine whether there were significant differences within a parcellation, followed by Tukey’s HSD to determine if there were significant pairwise differences. We performed FDR correction to control for multiple comparisons across the metrics and factors. To establish predictability, we performed dominance analysis, testing every possible permutation of the input variables and evaluating the contribution of each variable to multilinear models. For cross-validation analysis, to avoid spatial-autocorrelation effects we set the closest 75% of regions to be the training set and the furthest 25% of regions to be the validation set. Finally, we performed mediation analysis to determine the contribution of thickness and myelin to the relationship between the von Economo and Koskinas structural types and Mesulam’s hierarchy and the dMRI microstructural metrics.

### Reporting summary

Further information on research design is available in the Nature Portfolio Reporting Summary linked to this article.

## Supplementary information

Supplementary Materials

Reporting Summary

## Data Availability

The DTI, DKI, NODDI, and MAP-MRI cortical maps can be downloaded at https://github.com/ucsfncl/diffusion_neuromaps. HCP-YA data can be accessed and downloaded at https://www.humanconnectome.org. Volumetric PET images can be found at https://github.com/netneurolab/hansen_receptors or accessed using the neuromaps package: https://github.com/netneurolab/neuromaps. Other relevant cortical maps can also be found using the neuromaps package. BigBrain related data can be found using the BigBrainWarp package: https://github.com/caseypaquola/BigBrainWarp.

## Code availability

All code used in the analysis can be found at https://github.com/ucsfncl/diffusion_neuromaps.
