# Supplementary material for: Mapping the microstructure of human cerebral cortex in vivo with diffusion MRI
Source: Commun Biol. 2025 Jul 22;8:1088. doi: 10.1038/s42003-025-08523-9 (PMC12284103; doi:10.1038/s42003-025-08523-9)
Supplement: Supplementary file 1 — Supplementary Materials [file 42003_2025_8523_MOESM1_ESM.pdf]

Supplementary Material

Fig. S1: Intersubject coefficient of variation of cortical microstructure.

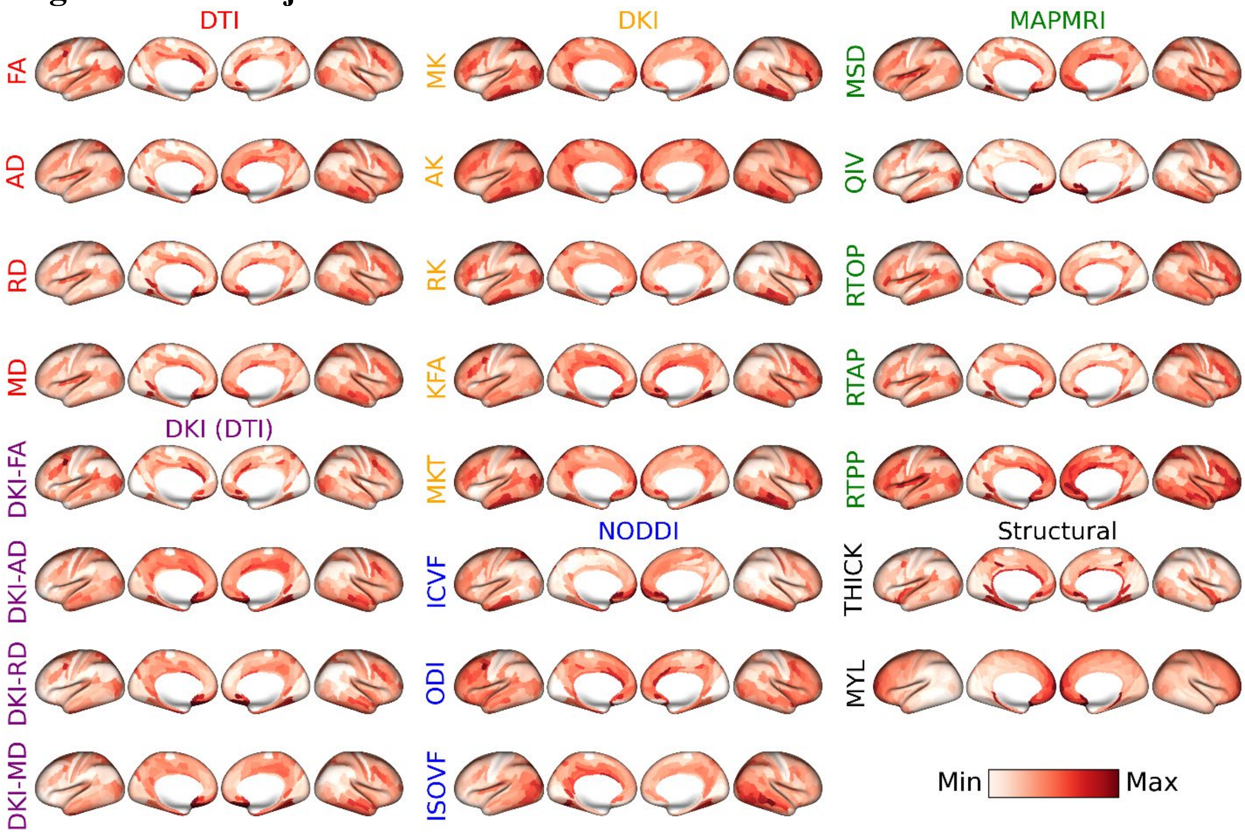

Microstructural maps were collected from each subject of the HCP-YA dataset; the variance across subjects was calculated; the measurement error, quantified via test-retest sessions, was corrected for; and the resulting between-subject standard deviation was divided by the group mean to derive the intersubject coefficient of variation (CoV). Many structural maps portray a gradient from low values in primary motor and sensory areas to high values in association regions.

Fig. S2: Laterality index of cortical microstructure.

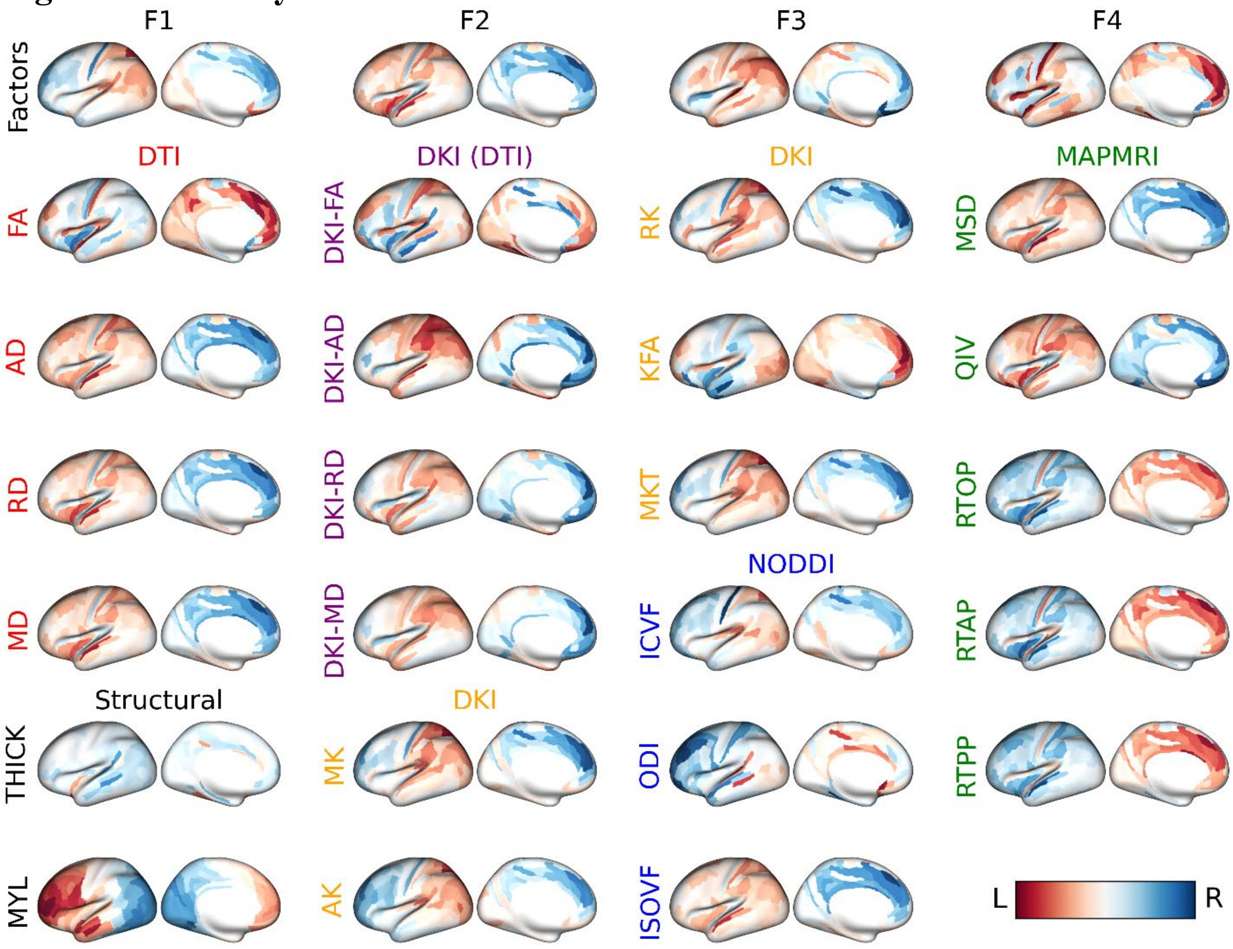

Microstructural maps were collected from the HCP-YA dataset for each subject, the group mean was taken, and the laterality index (LI) was computed for each metric. Laterality is computed as $\frac{x_{LH}-x_{RH}}{x_{LH}+x_{RH}}$, with Red indicating maximal left lateralization and blue indicating maximal right lateralization. The *top row* consists of the four explanatory factors: F1: diffusion kurtosis; F2: isotropic diffusion; F3: complex diffusion; F4: diffusion anisotropy. The columns below the top row consist of laterality maps of individual microstructural metrics.

Fig. S3: Alternative Dimensionality Reduction for the Cortical dMRI Microstructure

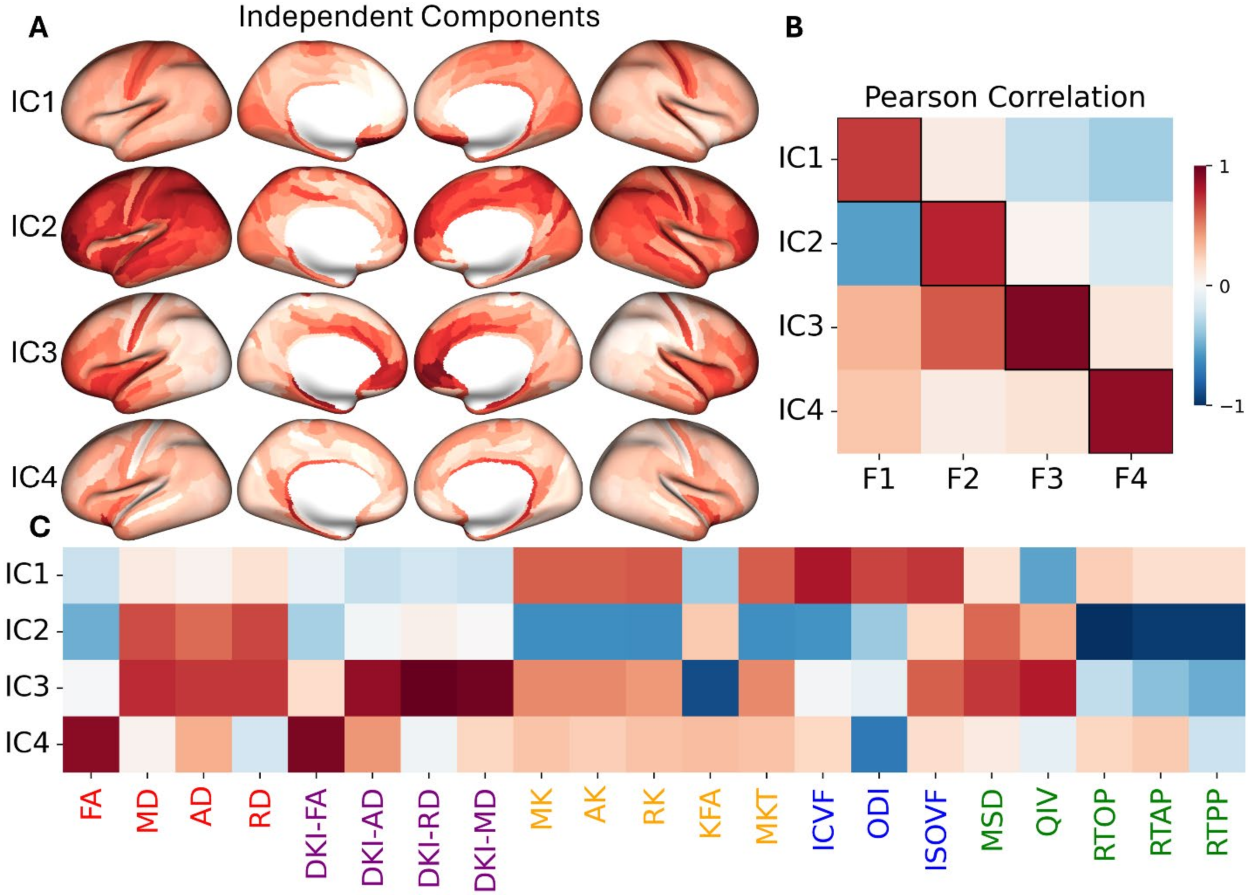

Alternative dimensionality reduction via independent component analysis (ICA) with four independent components (ICs) on the dMRI metrics. We have reordered ICs and flipped their sign to correspond with their identically numbered factor i.e. IC1 vs. F1. **(A)** The ICs bear strong resemblance to their corresponding factors. We note that IC2 and F2 as well as IC4 and F4 are negatively correlated. **(B)** Every IC was significantly correlated with their factor (IC1 vs. F1: r=0.703, p=0.0008; IC2 vs. F2: r=0.767, p=0.0004; IC3 vs. F3: r=0.935, p=0.0004; IC4 vs. F4: r=0.887, p=0.0008). Due to the differences between ICA and factor analysis, there was considerable bleed through, where F1 was associated with IC1, IC2, and IC3 and F2 was associated with IC2 and IC3. **(C)** The mixing matrix from ICA analysis shows how IC1 had strong positive associations with the kurtoses, neurite density (ICVF), neurite orientation dispersion index (ODI), and free water fraction (ISOVF); IC2 had strong positive associations with the DTI diffusivities and strong negative associations with the kurtoses and return probabilities, IC3 had strong positive associations with the DTI and DKI diffusivities, ISOVF, MSD, and QIV; IC4 has strong positive associations with FA and negative associations with ODI. We found IC1 to explain 26.1% of the variance, IC2 to account for 35.7% of the variance, IC3 to explain 40.6% of the variance, and IC4 to account for 21.0% of the variance.

Fig. S4: Structural Gradient Variation explained via dMRI metrics

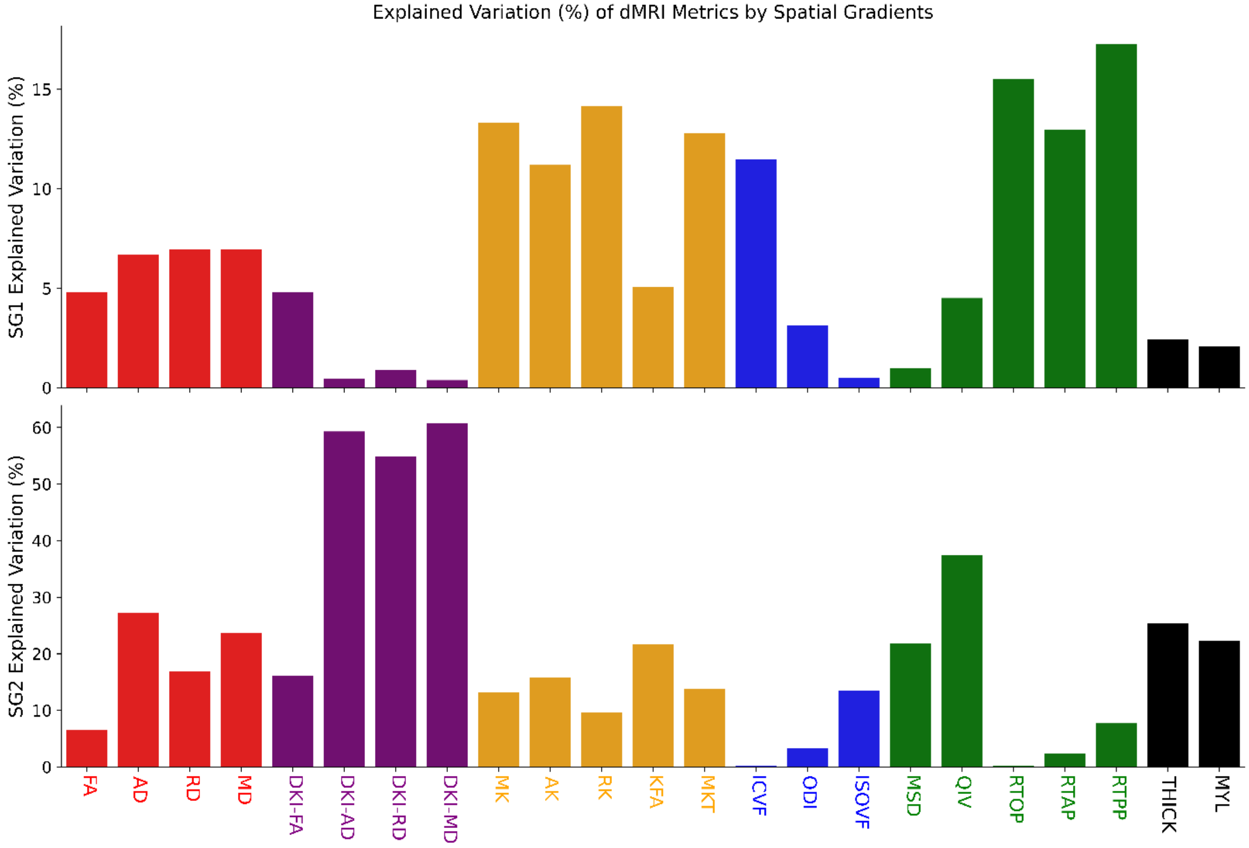

The percentage of variation of the first two structural gradients (SG1) and (SG2) that can be explained by each of the dMRI metrics, measured via the coefficient of determination.

Fig. S5: Divergence of cortical microstructure along the sensorimotor association axis.

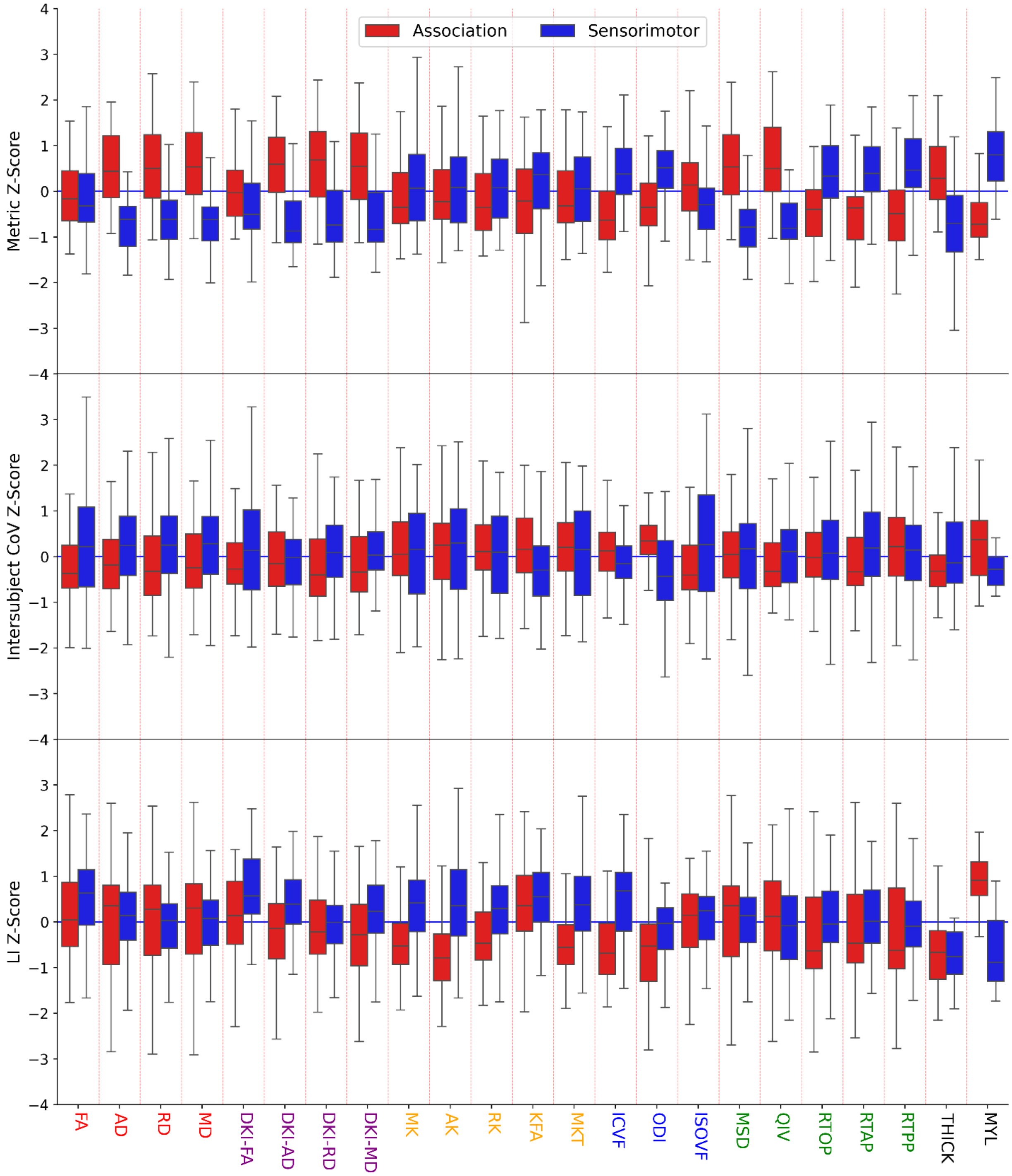

The divergence of microstructural values (top), intersubject coefficient of variation (CoV) (middle), and laterality index (LI) (bottom) along the sensorimotor-association (SA) axis. Association and sensorimotor regions are shown in red and blue respectively. The AD, RD

MD, DKI-AD, DKI-RD, DKI-MD, KFA, ICVF, ODI, MSD, QIV, RTOP, RTAP, RTPP, thickness and myelin values; the RD, KFA, ICVF, ODI, ISOVF, and intersubject CoVs, and the DKI-FA, DKI-AD, DKI-MD, MK, AK, RK, MKT, ICVF, ODI, and myelin LIs show statistically significant divergence (FDR-corrected, Welch t-test, $P < 0.05$).

Fig. S6: Significant Correlations between Cortical dMRI Microstructure and SA Axis components

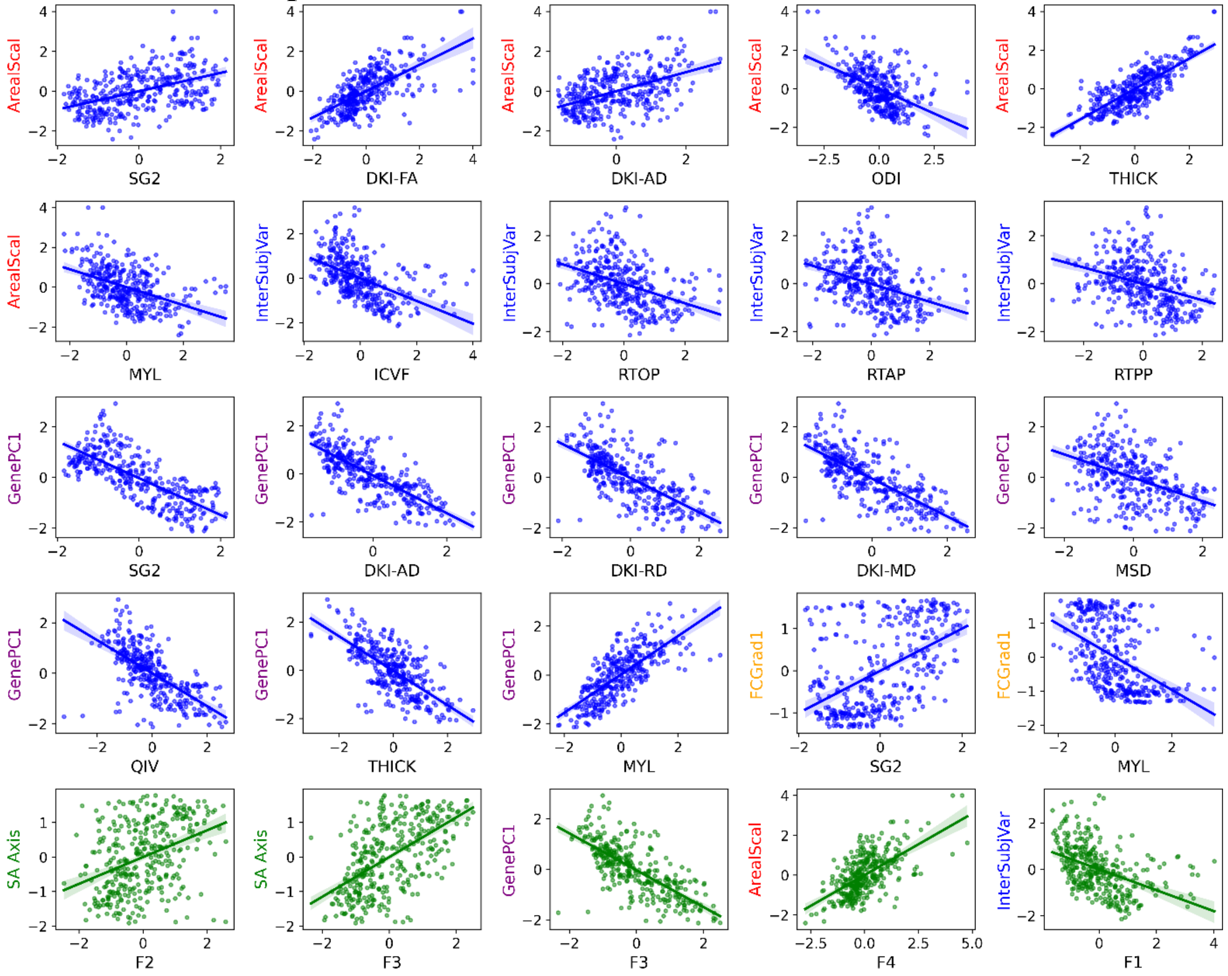

Scatterplots of the significant correlations, after FDR correction, between cortical dMRI microstructural metrics including the factors (F1-F4) and SG2 (second structural gradient) and several sensorimotor-association (SA) axis components: areal scaling (ArealScal), functional intersubject variability (InterSubjVar), the first principal component of gene expression (GenePC1), and the first gradient of functional connectivity (FCGrad1). For abbreviations of cortical dMRI metrics and factors on the x-axes, please consult Table S1.

Fig. S7: Stratification of cortical microstructure across von Economo and Koskinas cell types.

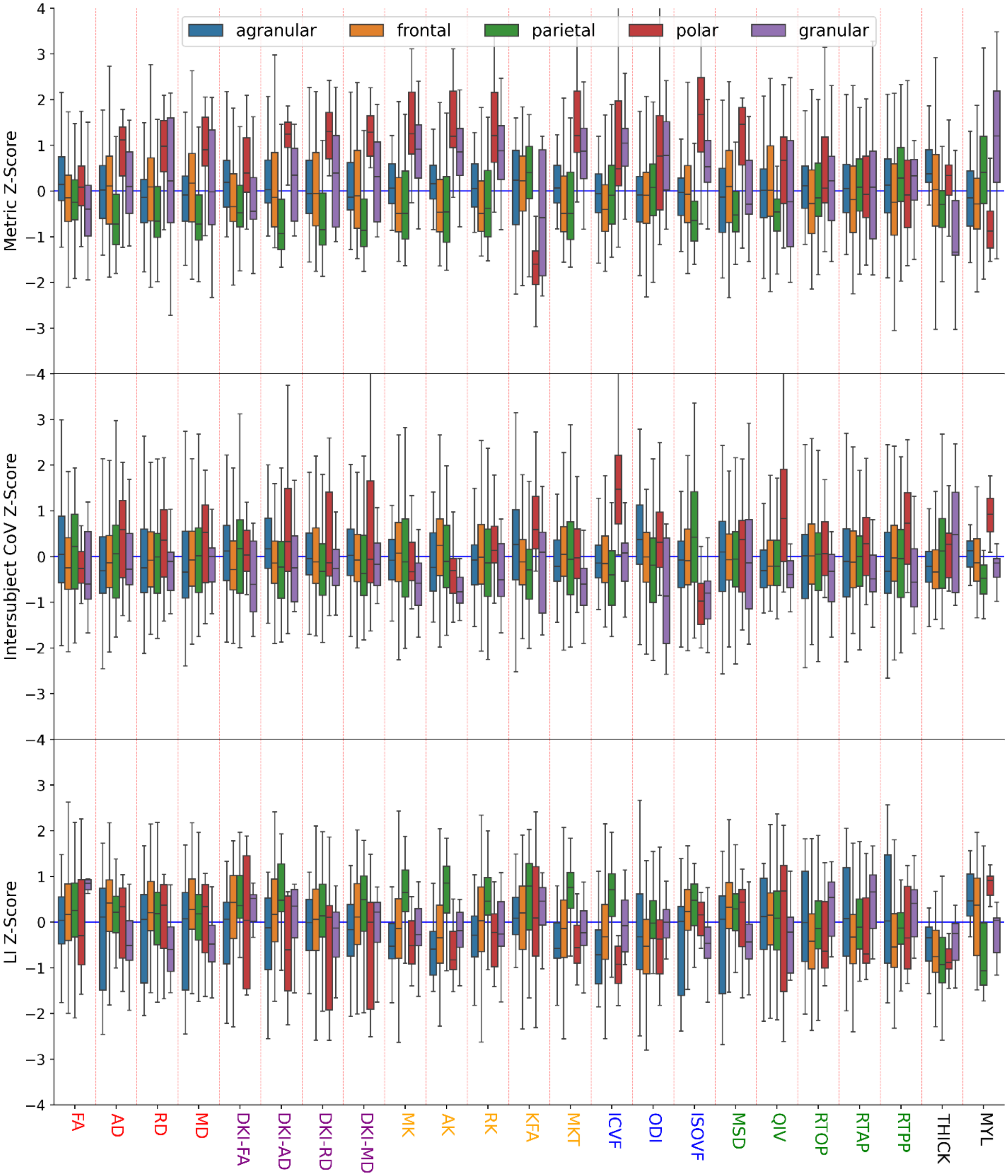

Microstructural values (top), intersubject coefficient of variation (CoV) (middle), and laterality index (LI) (bottom) stratified by the von Economo and Koskinas cell structural types via atlas-based parcellation. Every metric apart from FA showed statistically significant stratification

across the von Economo cell types (FDR-corrected, one-way ANOVA, $P < 0.05$). The DKI-FA, DKI-AD, DKI-RD, DKI-MD, MK, AK, KFA, MKT, ICVF, ODI, ISOVF, QIV, RTPP, thickness, and myelin intersubject CoV as well as the DKI-AD, MK, AK, RK, MKT, ICVF, ISOVF, and myelin LI showed statistically significant stratification across the von Economo cell types (FDR-corrected, one-way ANOVA, $P < 0.05$).

Fig. S8: Stratification of cortical microstructural across Mesulam's hierarchy of laminar differentiation.

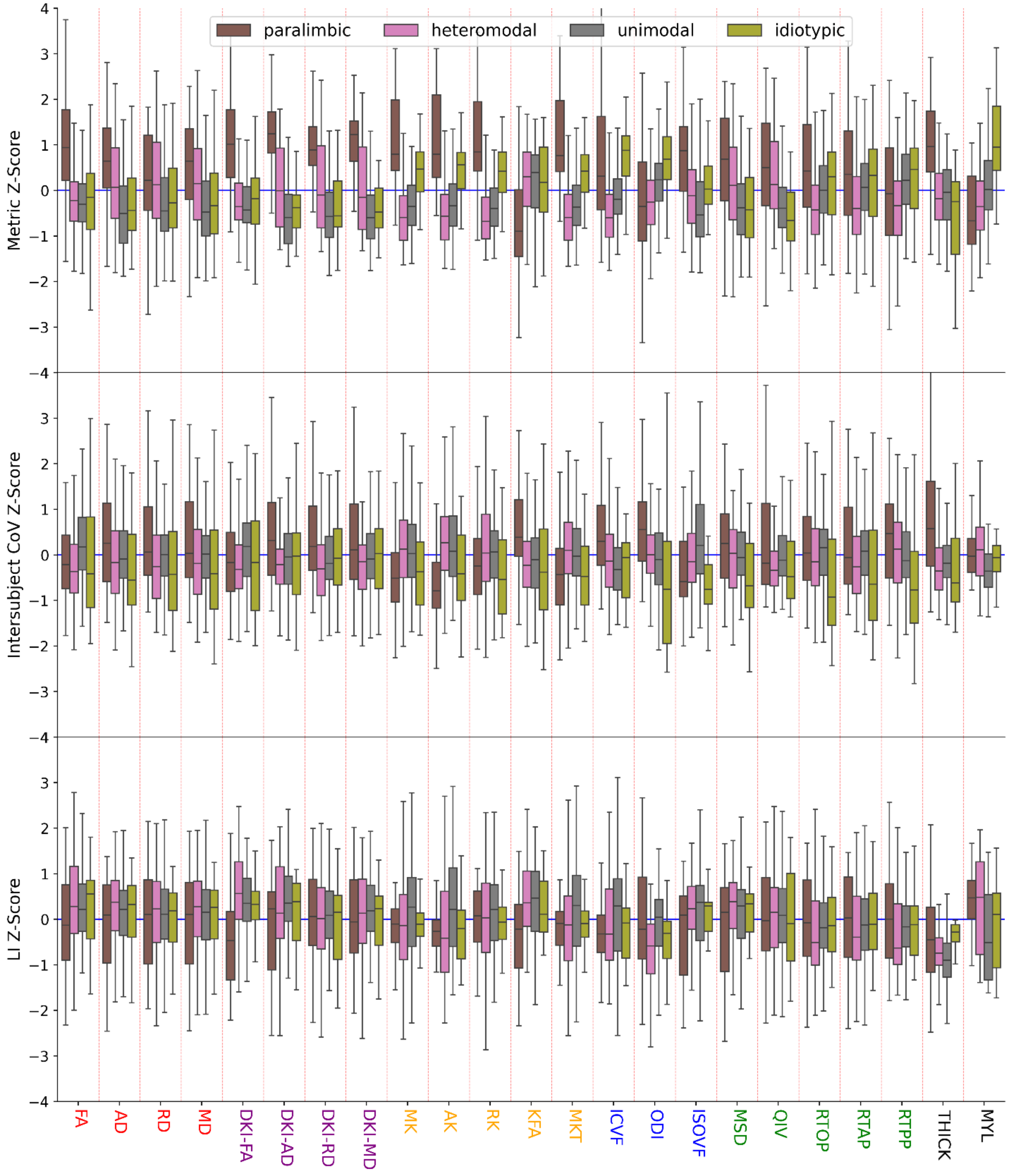

Microstructural values (top), intersubject coefficient of variation (CoV) (middle), and laterality index (LI) (bottom) stratified by Mesulam's hierarchy of laminar differentiation via atlas-based parcellation. Every microstructural value and intersubject CoV and specifically the DKI-FA, KFA, ODI, and myelin LIs showed statistically significant stratification across laminar differentiation (FDR-corrected, one-way ANOVA, $P < 0.05$)

Fig. S9: Stratification of cortical microstructural across functional networks.

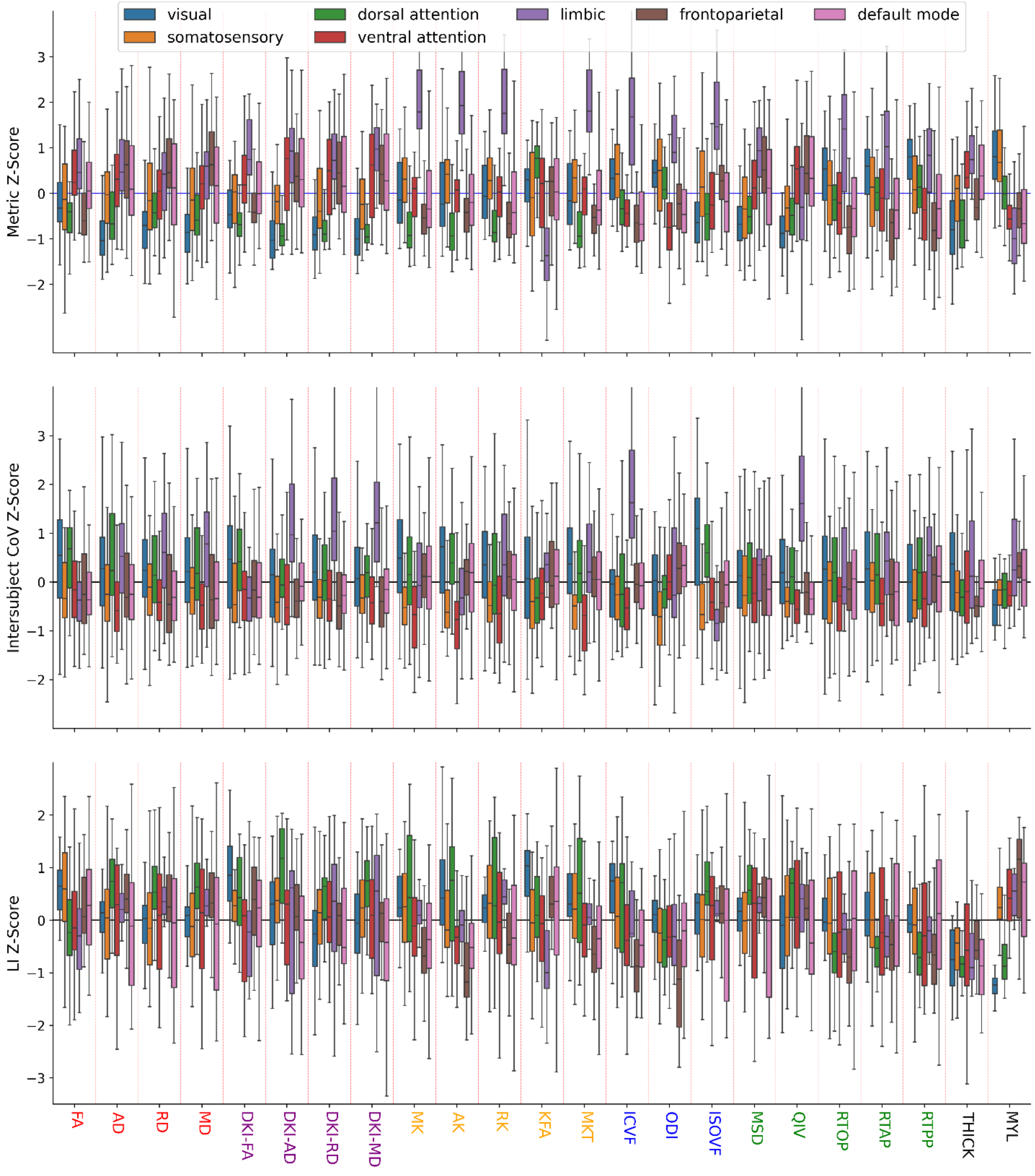

Microstructural values (top), intersubject coefficient of variation (CoV) (middle), and laterality index (LI) (bottom) stratified by functional networks (Yeo parcellation). Every microstructural value and intersubject CoV and most LIs show statistically significant stratification across functional networks (FDR-corrected, one-way ANOVA, $P < 0.05$).

Fig. S10: Cortical microstructure variation explained via multiple organizational hierarchies.

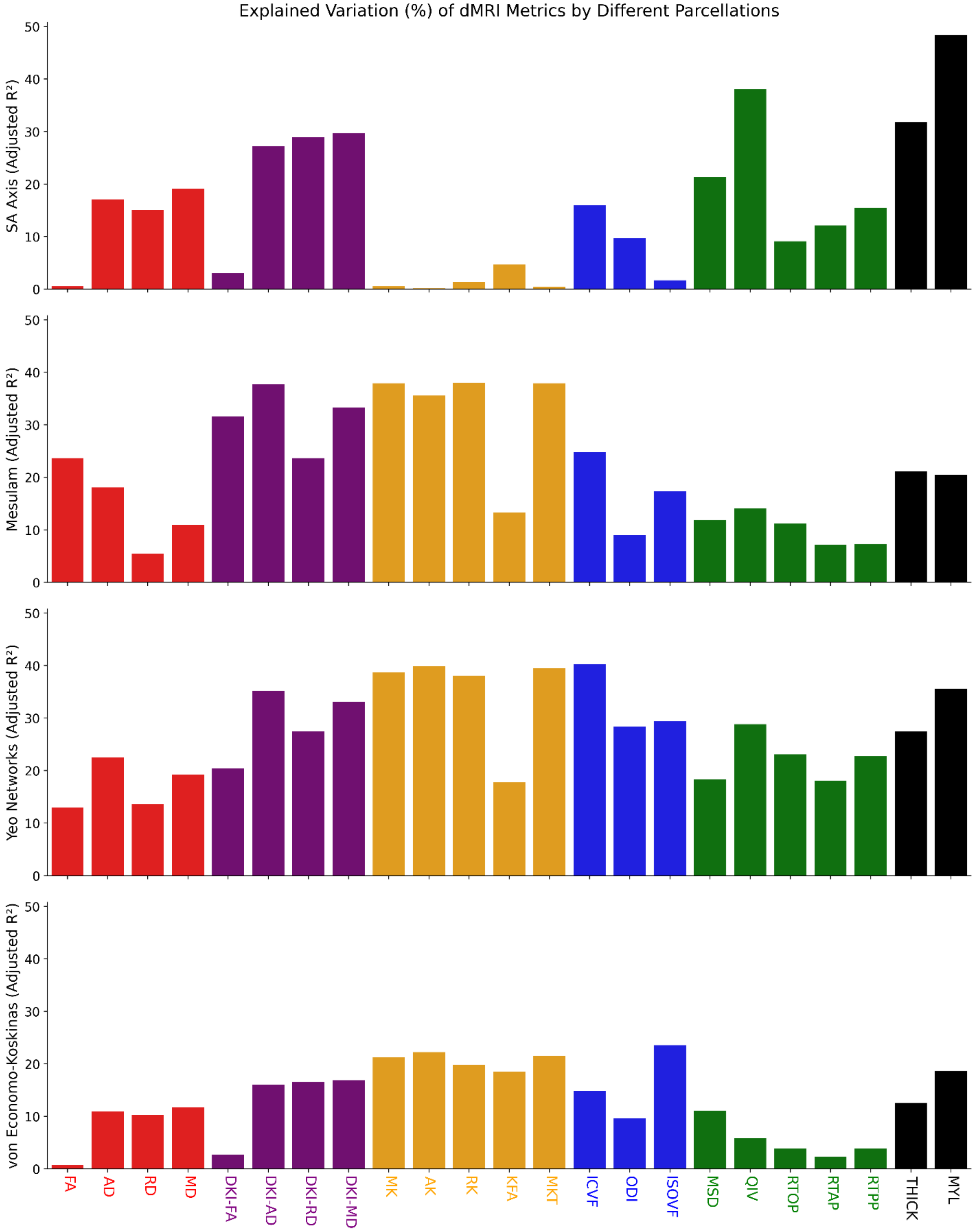

The percentage of variation that can be explained by the sensorimotor-association (SA) axis, Mesulam's hierarchy, Yeo networks, and von Economo networks measured via the adjusted coefficient of determination.

Fig. S11: Multivariate MEG power and timescale prediction.

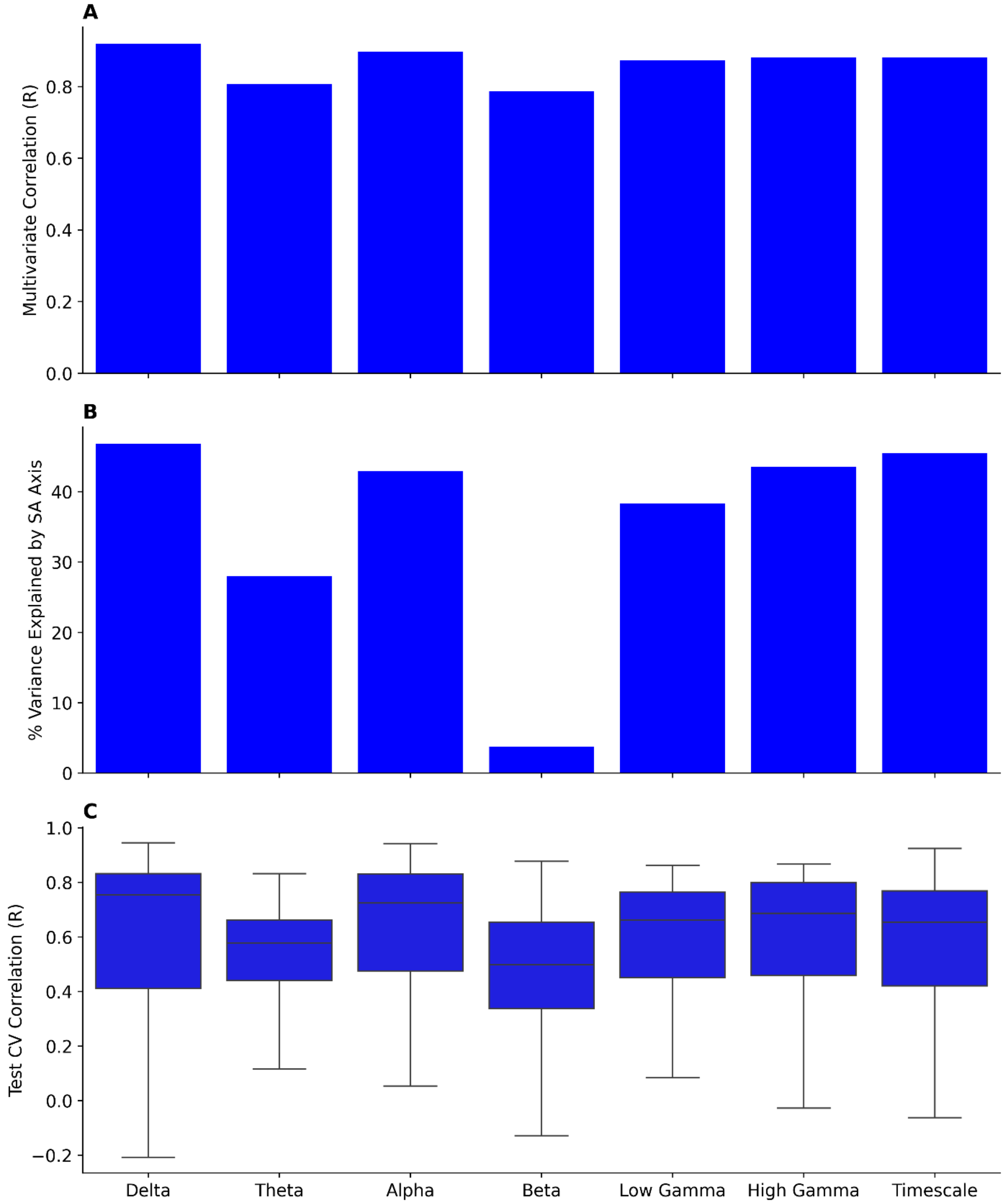

(A) The correlation coefficient from multiple linear regression of MEG power prediction for each frequency band as well as timescale. **(B)** The percentage of the variance of the prediction explained by divergence along the sensorimotor-association axis. **(C)** The test cross-validation (CV) correlation coefficient of MEG power prediction for each frequency band as well as

timescale. Distance-dependent CV was performed for each region in the Glasser parcellation by setting the test set to the closest 90 regions and the training set to the furthest 270 regions.

Fig. S12: Cortical microstructure shapes neurotransmitter receptor densities.

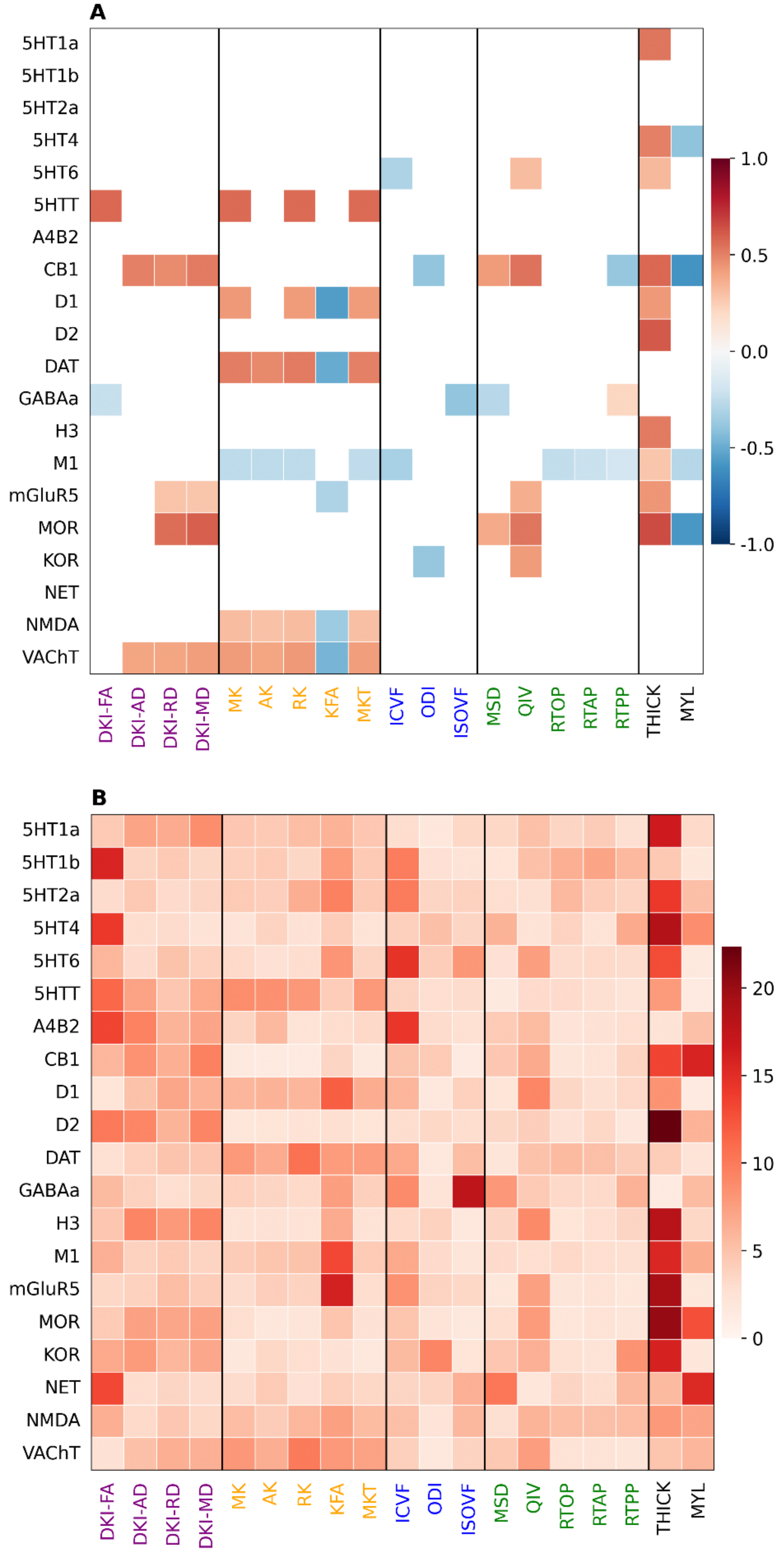

(A) Pearson correlation coefficients between normalized receptor/transporter densities measured by PET and structural metrics with only statically significant relationships shown. **(B)** Dominance analysis showing the relative contribution of every microstructural metric to a linear model for each receptor or transporter. See Table S2 for abbreviations of all neurotransmitter receptors/transporters.

Fig. S13: Multivariate neurotransmitter receptor density prediction.

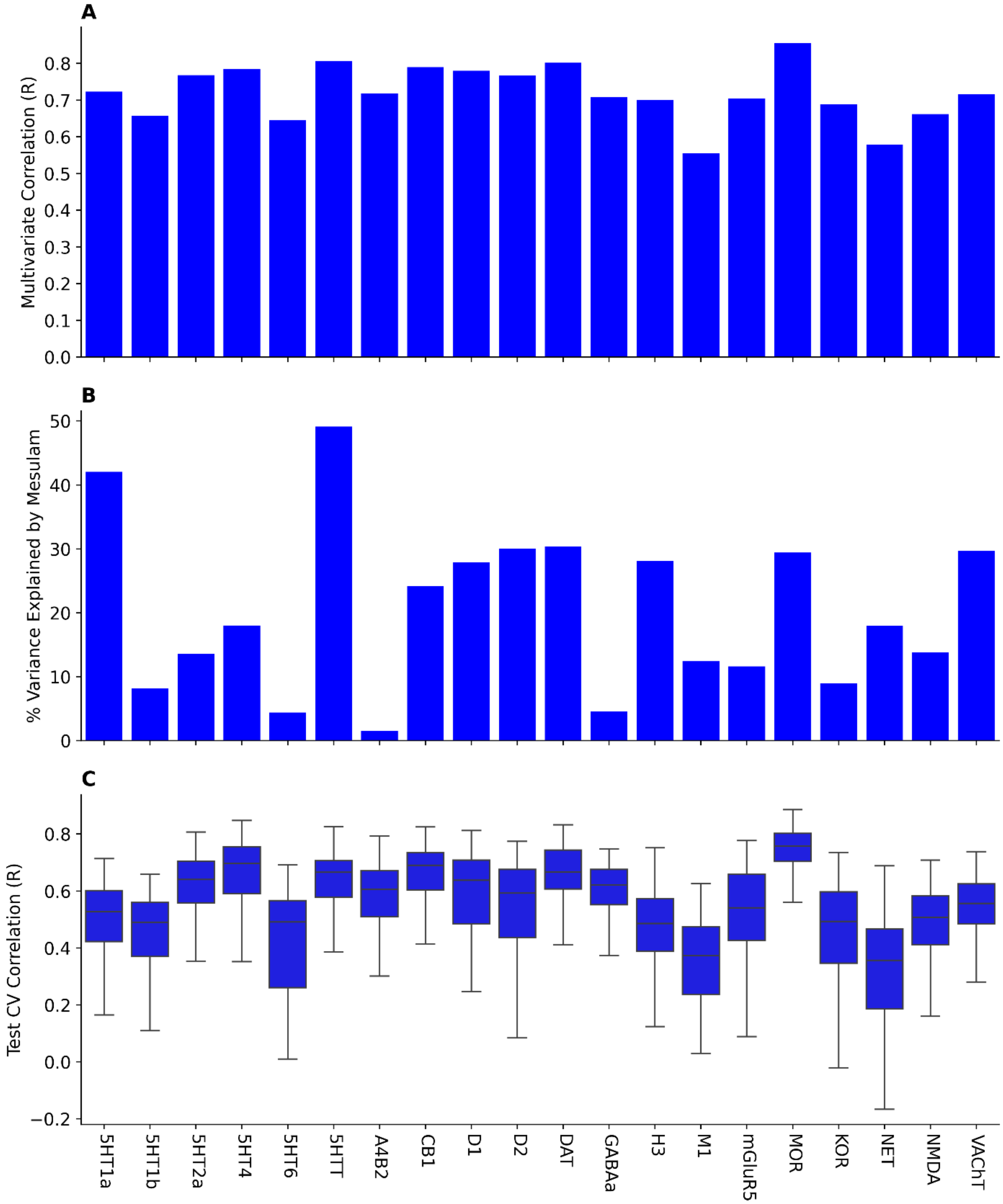

(A) The correlation coefficient from multiple linear regression of neurotransmitter receptor density estimation. **(B)** The percentage of the variance of the prediction explained by stratification across Mesulam's hierarchy of laminar differentiation. **(C)** The test cross-validation (CV) correlation coefficient of neurotransmitter receptor density prediction.

Distance-dependent CV was performed for each region in the Glasser parcellation by setting the test set to the closest 90 regions and the training set to the furthest 270 regions.

Fig. S14: Microstructural test-retest coefficient of variation.

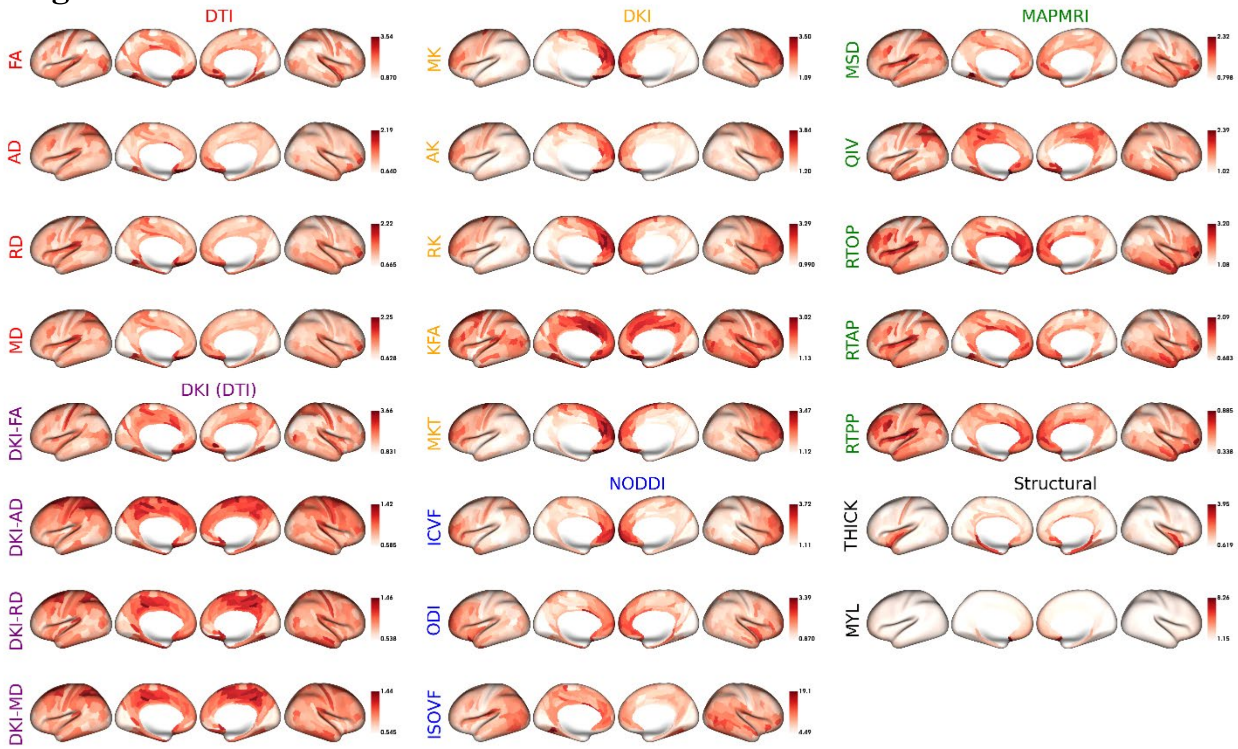

The test-retest coefficient of variation (CoV) for microstructural values across Glasser parcels computed using the test-retest portion of HCP-YA dataset (n=38).

Fig. S15: The intraclass correlation of cortical microstructure.

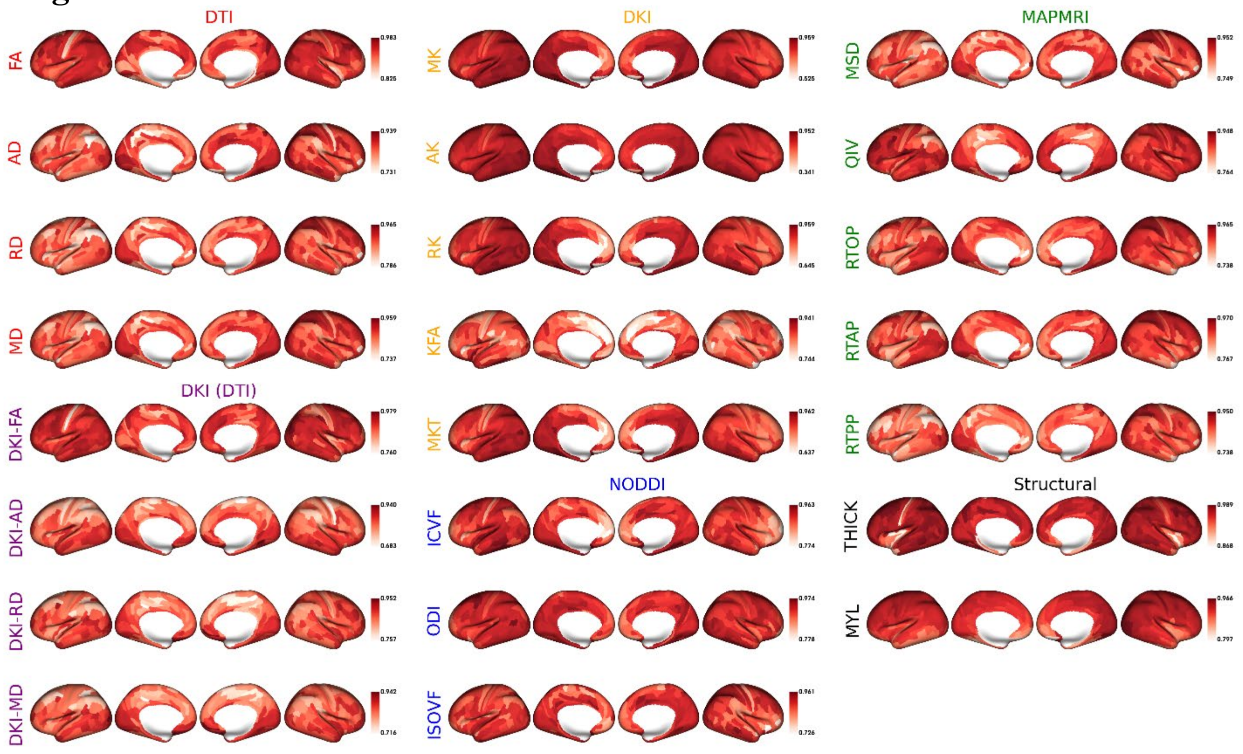

The two-way mixed, single measures, absolute agreement intraclass correlation coefficient (ICC) for the cortical microstructural metrics across Glasser parcels. The within-subject variance was found via the test-retest portion of the HCP-YA dataset (n=38) and the between-subject variance was found via the HCP-YA dataset (n=962).

Fig. S16: Replication of cortical microstructural maps in the MGH-USC data.

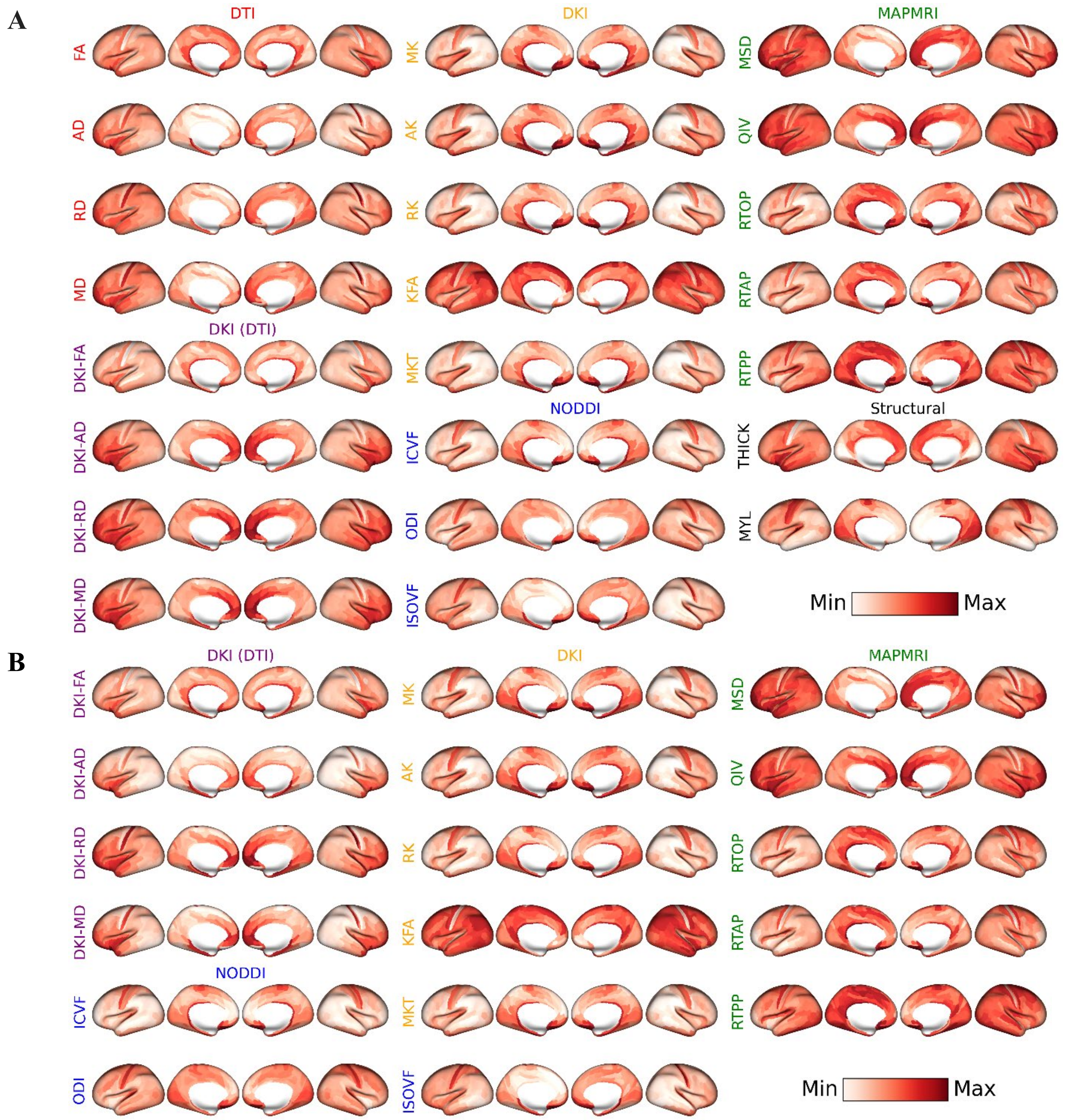

Group-averaged microstructural maps from the MGH-USC dataset using **(A)** all four shells at b=1000, 3000, 5000 & 10000 s/mm^2 (except for the first four DTI maps at b=1000 s/mm^2 only); and **(B)** only the b=1000 & 3000 s/mm^2 shells to match same shells of the HCP-YA dataset. As dMRI microstructural values span several orders of magnitude (1e-9 to 1e6), we display group-averaged microstructural values standardized to zero mean and unit standard deviation across the cortex for ease of comparison across various microstructural metrics.

Fig. S17: Cortical microstructure in the HCP-YA versus MGH-USC datasets.

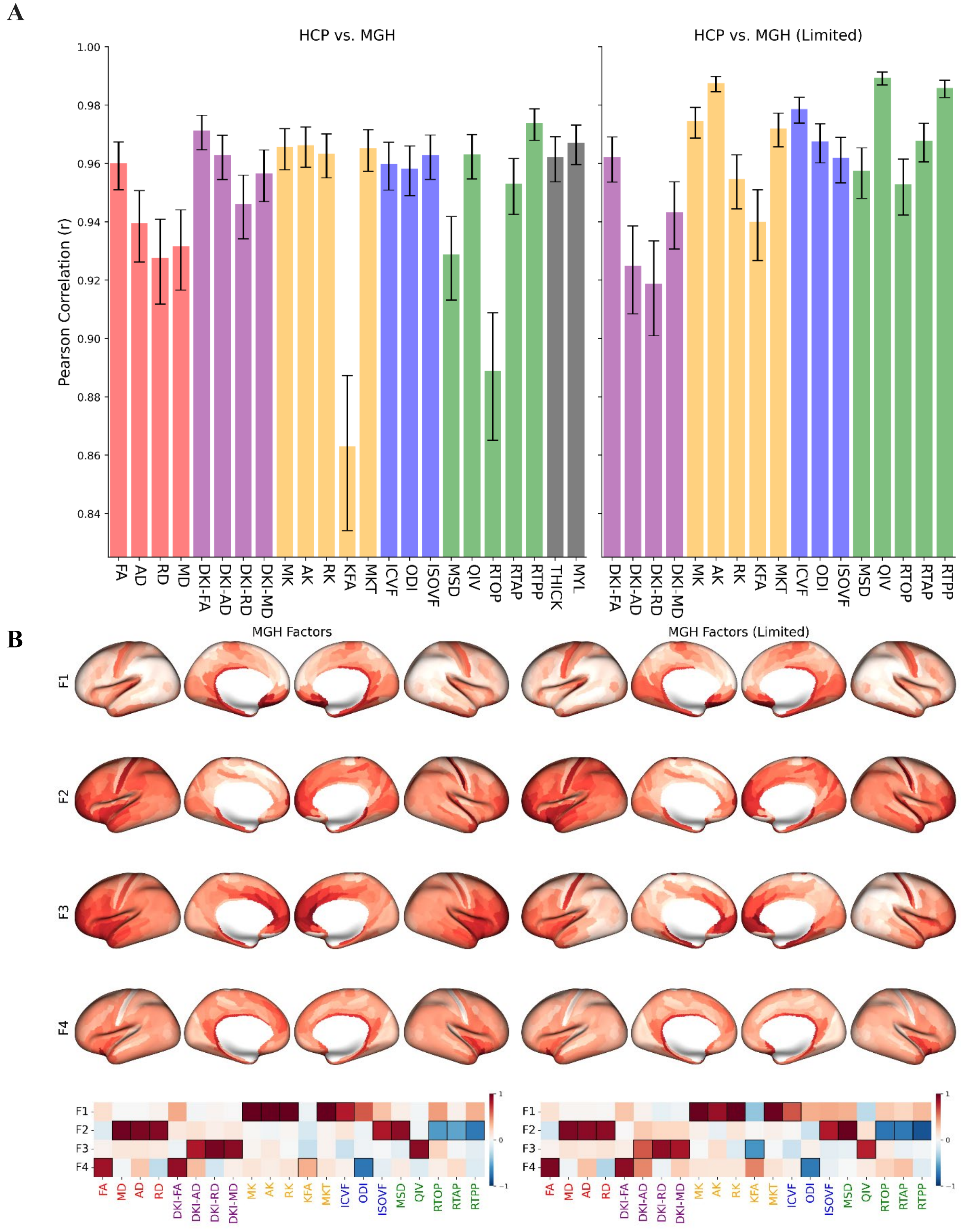

(**A**) Correlation coefficients with 95% confidence intervals of individual cortical dMRI metrics of the HCP-YA (HCP) data versus (*left barplot*) all four shells (b=1000, 3000, 5000 & 10000 s/mm^2) of the MGH-USC (MGH) data (except for the first four DTI metrics at b=1000 s/mm^2 only); and (*right barplot*) only the b=1000 & 3000 s/mm^2 shells of the MGH-USC data to match the same shells of the HCP-YA dataset. **(B)** The cortical maps of the four explanatory factors and their loadings across the individual dMRI metrics for (*left*) all four shells of the MGH-USC dataset and (*right*) for the b=1000 & 3000 s/mm^2 shells only both closely match the corresponding four explanatory factors of the HCP-YA dataset shown in Figure 1.

Fig. S18: Cortical microstructure: HCP-YA vs MGH-USC datasets.

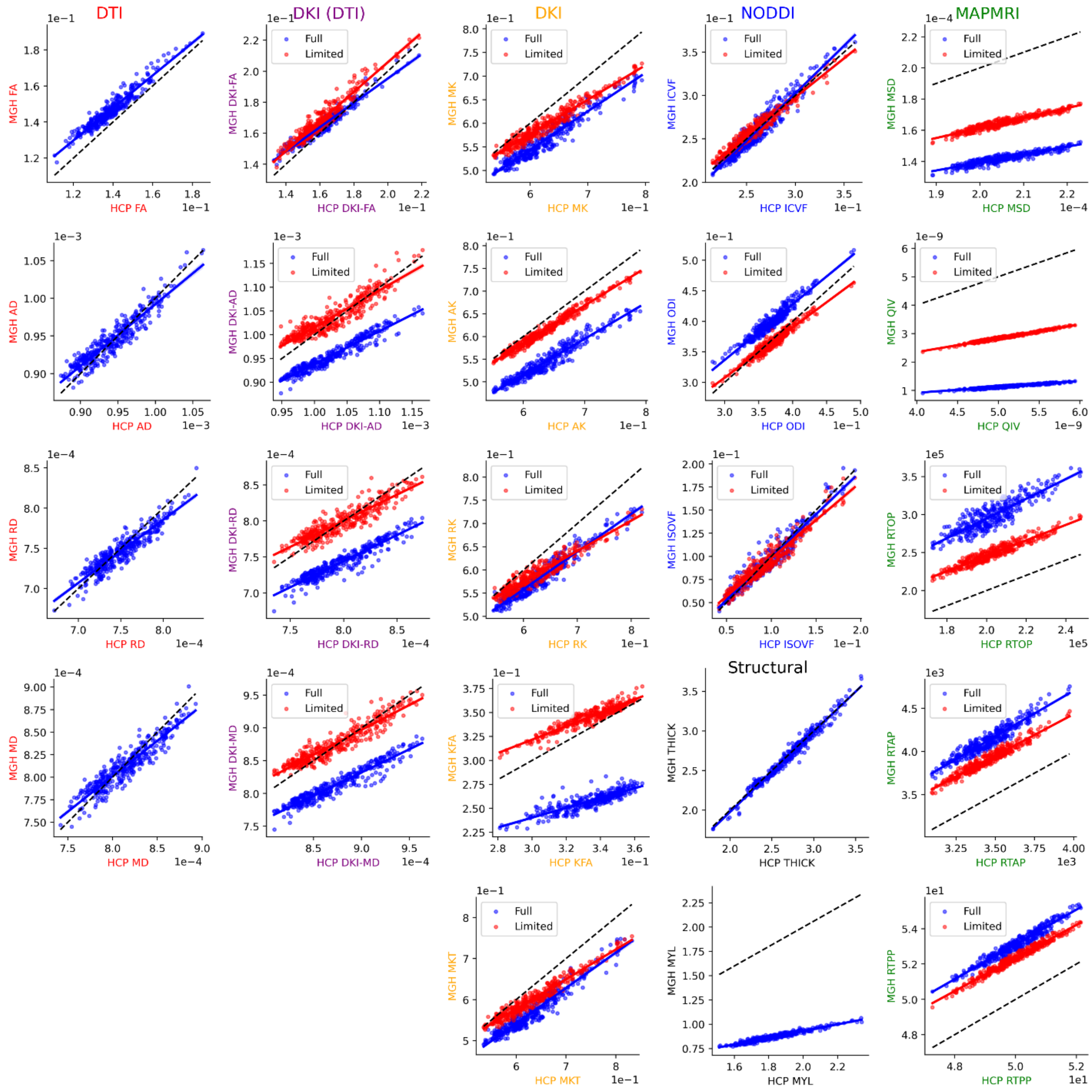

Scatterplots of group-averaged cortical dMRI metrics across the Glasser parcellation (360 regions) of the HCP-YA (HCP) maps versus MGH-USC (MGH) maps for (*Full in blue*) all four shells (b=1000, 3000, 5000 & 10000 s/mm^2) of the MGH-USC data (except for the first four DTI metrics at b=1000 s/mm^2 only); and (*Limited in red*) only the b=1000 & 3000 s/mm^2 shells of the MGH-USC data to match the same shells of the HCP-YA dataset.

Table S1: Abbreviations for Cortical dMRI Metrics and Factors

Acronym	Full Name	Signal Model/Tissue Representation
FA	Fractional Anisotropy	Diffusion Tensor Imaging (DTI)
AD	Axial Diffusivity	Diffusion Tensor Imaging (DTI)
RD	Radial Diffusivity	Diffusion Tensor Imaging (DTI)
MD	Mean Diffusivity	Diffusion Tensor Imaging (DTI)
DKI-FA	Fractional Anisotropy	Diffusion Kurtosis Imaging (DKI)
DKI-AD	Axial Diffusivity	Diffusion Kurtosis Imaging (DKI)
DKI-RD	Radial Diffusivity	Diffusion Kurtosis Imaging (DKI)
DKI-MD	Mean Diffusivity	Diffusion Kurtosis Imaging (DKI)
MK	Mean Kurtosis	Diffusion Kurtosis Imaging (DKI)
AK	Axial Kurtosis	Diffusion Kurtosis Imaging (DKI)
RK	Radial Kurtosis	Diffusion Kurtosis Imaging (DKI)
MKT	Mean Kurtosis Tensor	Diffusion Kurtosis Imaging (DKI)
KFA	Kurtosis Fractional Anisotropy	Diffusion Kurtosis Imaging (DKI)
ICVF	Intracellular Volume Fraction (neurite density)	Neurite Orientation Dispersion and Density Imaging (NODDI)
ODI	(neurite) Orientation Dispersion Index	Neurite Orientation Dispersion and Density Imaging (NODDI)
ISOVF	Isotropic Volume Fraction (free water fraction)	Neurite Orientation Dispersion and Density Imaging (NODDI)
MSD	Mean Squared displacement	Mean Apparent Propagator MRI (MAP-MRI)
QIV	Q-space Inverse Variance	Mean Apparent Propagator MRI (MAP-MRI)
RTOP	Return to Origin Probability	Mean Apparent Propagator MRI (MAP-MRI)
RTAP	Return to Axis Probability	Mean Apparent Propagator MRI (MAP-MRI)
RTPP	Return to Plane Probability	Mean Apparent Propagator MRI (MAP-MRI)

F1	Factor 1: Kurtoses/Neurite Density/Cellularity	Factor Analysis
F2	Factor 2: Diffusivities/Free Water	Factor Analysis
F3	Factor 3: Complex Diffusivity/Extracellular Volume Fraction	Factor Analysis
F4	Factor 4: Anisotropies/ Neurite Orientation Dispersion	Factor Analysis

We provide full names for the cortical microstructural metrics that we quantify from diffusion MRI: FA, AD, RD, and MD from DTI; DKI-FA, DKI-AD, DKI-RD, DKI-MD, MK, AK, RK, MKT, KFA from DKI; ICVF, ODI, and ISOVF from NODDI, MSD, QIV, RTOP, RTAP, and RTPP. We also include the same for the factors (F1-F4).

Table S2: Abbreviations for Neurotransmitter Receptors/Transporters

**Acronym**	**Full Name**	**Neurotransmitter**
5HT1a	5-Hydroxytryptamine Receptor 1A	Serotonin
5HT1b	5-Hydroxytryptamine Receptor 1B	Serotonin
5HT2a	5-Hydroxytryptamine Receptor 2A	Serotonin
5HT4	5-Hydroxytryptamine Receptor 4	Serotonin
5HT6	5-Hydroxytryptamine Receptor 6	Serotonin
5HTT	Serotonin Transporter	Serotonin
A4B2	Alpha-4 Beta-2 Nicotinic Acetylcholine Receptor	Acetylcholine
CB1	Cannabinoid Receptor 1	Cannabinoids
D1	Dopamine Receptor D1	Dopamine
D2	Dopamine Receptor D2	Dopamine
DAT	Dopamine Transporter	Dopamine
GABAa	Gamma-aminobutyric acid Type A Receptor	Gamma-aminobutyric acid (GABA)
H3	Histamine Receptor H3	Histamine
M1	Muscarinic Acetylcholine Receptor M1	Acetylcholine
mGluR5	Metabotropic Glutamate Receptor 5	Glutamate
MOR	Mu-Opioid Receptor	Opioids
KOR	Kappa-Opioid Receptor	Opioids
NET	Norepinephrine Transporter	Norepinephrine
NMDA	N-Methyl-D-Aspartate Receptor	Glutamate
VAChT	Vesicular Acetylcholine Transporter	Acetylcholine

We provide full names for the neurotransmitter receptor/transporter density distributions that we investigate: 5HT1a, 5HT1b, 5HT2a, 5HT4, 5HT6, 5HTT, A4B2, CB1, D1, D2, DAT, GABAa, H3, M1, mGluR5, MOR, KOR, NET, NMDA, VAChT. We also include their respective neurotransmitter.

Table S3: Significant Pairwise Differences across Mesulam's Hierarchy of Laminar Differentiation

Factor	Group 1	Group 2	q-statistic	p-value
F1	paralimbic	heteromodal	1.50	6.0e-14
F1	paralimbic	unimodal	1.28	6.0e-14
F1	paralimbic	idiotypic	0.55	3.5e-03
F1	heteromodal	idiotypic	-0.95	7.6e-10
F1	unimodal	idiotypic	-0.73	4.6e-06
F2	paralimbic	unimodal	0.60	2.8e-04
F2	heteromodal	unimodal	0.57	3.4e-05
F2	heteromodal	idiotypic	0.44	4.7e-02
F3	paralimbic	heteromodal	0.83	1.7e-09
F3	paralimbic	unimodal	1.44	6.0e-14
F3	paralimbic	idiotypic	1.44	6.0e-14
F3	heteromodal	unimodal	0.61	1.8e-07
F3	heteromodal	idiotypic	0.61	3.0e-04
F4	paralimbic	heteromodal	1.01	1.5e-12
F4	paralimbic	unimodal	1.27	6.0e-14
F4	paralimbic	idiotypic	1.42	6.7e-14
F4	heteromodal	idiotypic	0.42	3.5e-02
thickness	paralimbic	heteromodal	1.02	9.8e-13
thickness	paralimbic	unimodal	1.01	3.6e-12
thickness	paralimbic	idiotypic	1.59	6.0e-14
thickness	heteromodal	idiotypic	0.56	1.9e-03
thickness	unimodal	idiotypic	0.58	1.3e-03
myelin	paralimbic	unimodal	-0.51	9.8e-04
myelin	paralimbic	idiotypic	-1.42	7.6e-14
myelin	heteromodal	unimodal	-0.50	7.9e-05
myelin	heteromodal	idiotypic	-1.41	6.0e-14
myelin	unimodal	idiotypic	-0.91	7.9e-08

The significant pairwise differences found for the factors (F1-F4), cortical thickness, and cortical myelination across Mesulam's hierarchy of laminar differentiation.

Table S4: Significant Pairwise Differences across the von Economo and Koskinas Structural Types

Factor/Metric	Group 1	Group 2	q-statistic	p-value
F1	agranular	frontal	0.40	2.1e-02
F1	agranular	polar	-1.26	1.3e-07
F1	agranular	granular	-0.74	1.7e-02
F1	frontal	polar	-1.66	1.5e-13
F1	frontal	granular	-1.14	4.9e-06
F1	parietal	polar	-1.58	3.9e-12
F1	parietal	granular	-1.06	6.1e-05
F2	agranular	polar	-0.72	1.9e-02

F2	frontal	parietal	0.59	4.5e-05
F2	parietal	polar	-0.99	1.6e-04
F3	agranular	parietal	0.79	2.4e-06
F3	agranular	polar	-0.75	8.3e-03
F3	frontal	parietal	0.66	1.2e-06
F3	frontal	polar	-0.88	2.3e-04
F3	parietal	polar	-1.54	6.3e-11
F3	parietal	granular	-0.88	2.5e-03
thickness	agranular	frontal	0.47	6.1e-03
thickness	agranular	parietal	0.89	1.2e-07
thickness	agranular	granular	1.51	3.0e-08
thickness	frontal	parietal	0.42	6.0e-03
thickness	frontal	granular	1.04	8.6e-05
thickness	parietal	polar	-0.63	3.2e-02
thickness	polar	granular	1.26	2.4e-04
myelin	agranular	parietal	-0.61	3.9e-04
myelin	agranular	granular	-1.34	4.5e-07
myelin	frontal	parietal	-0.75	1.0e-08
myelin	frontal	granular	-1.47	1.5e-09
myelin	parietal	polar	1.20	2.5e-07
myelin	parietal	granular	-0.73	1.7e-02
myelin	polar	granular	-1.93	4.0e-10

The significant pairwise differences found for the factors (F1-F4), cortical thickness, and cortical myelination across the von Economo and Koskinas structural types.

Table S5: The Proportion of dMRI Microstructure Mediated by Cortical Thickness and Myelin

Proportion Mediated (%)								
	Cortical Thickness				Cortical Myelination			
**Mesulam**	**F1**	**F2**	**F3**	**F4**	**F1**	**F2**	**F3**	**F4**
Idiotypic	28.8	7.0	36.6	55.3	76.3	57.8	57.6	11.6
Unimodal	3.2	0.3	5.3	12.4	3.3	1.7	3.0	0.05
Heteromodal	2.4	0.5	22.0	47.2	17.3	22.5	17.0	22.7
Paralimbic	13.8	7.0	25.6	38.9	13.0	20.8	15.1	2.6
	Cortical Thickness				Cortical Myelination			
**Von Economo**	**F1**	**F2**	**F3**	**F4**	**F1**	**F2**	**F3**	**F4**
Granular	-0.5	-1.2	-12.9	13.5	59.9	3.9	-14.7	48.4
Polar	1.8	3.1	16.7	-3.9	-22.2	18.7	37.7	-4.0
Parietal	3.2	2.8	15.3	11.4	-22.2	10.2	19.3	43.1
Frontal	-1.0	3.3	-35.0	60.2	18.3	27.3	-10.0	42.8
Agranular	-17.4	-13.6	16.8	10.5	33.3	-11.3	61.5	10.9

The proportion of the relationship (%) between the factors (F1-F4) and each of Mesulam's hierarchy and each of the von Economo and Koskinas structural types that is mediated by cortical thickness and myelination. Most relationships are mildly to moderately mediated by cortical thickness and myelination.

Table S6: Significant Pairwise Differences across the Yeo Functional Networks

Factor/Metric	Group 1	Group 2	q-statistic	p-value
F1	visual	dorsal attention	0.74	3.4e-05
F1	visual	limbic	-1.87	0.0
F1	visual	frontoparietal	0.62	1.3e-03
F1	somatosensory	dorsal attention	0.92	4.1e-08
F1	somatosensory	ventral attention	0.54	8.4e-03
F1	somatosensory	limbic	-1.68	0.0
F1	somatosensory	frontoparietal	0.81	4.3e-06
F1	somatosensory	default mode	0.57	3.8e-04
F1	dorsal attention	limbic	-2.61	0.0
F1	ventral attention	limbic	-2.22	0.0
F1	limbic	frontoparietal	2.49	0.0
F1	limbic	default mode	2.25	0.0
F2	visual	somatosensory	-0.74	5.5e-04
F2	visual	ventral attention	-1.05	5.6e-07
F2	visual	limbic	-0.88	9.3e-04
F2	visual	frontoparietal	-1.46	4.8e-13
F2	visual	default mode	-0.94	2.2e-07
F2	somatosensory	frontoparietal	-0.72	2.0e-03
F2	dorsal attention	frontoparietal	-0.96	1.6e-05
F2	frontoparietal	default mode	0.52	3.6e-02

F3	visual	somatosensory	-0.61	2.6e-03
F3	visual	ventral attention	-1.31	1.0e-12
F3	visual	limbic	-1.43	2.2e-11
F3	visual	frontoparietal	-1.22	5.9e-11
F3	visual	default mode	-1.30	0.0
F3	somatosensory	dorsal attention	0.56	1.3e-02
F3	somatosensory	ventral attention	-0.69	6.9e-04
F3	somatosensory	limbic	-0.82	5.3e-04
F3	somatosensory	frontoparietal	-0.60	6.1e-03
F3	somatosensory	default mode	-0.69	5.5e-05
F3	dorsal attention	ventral attention	-1.25	4.1e-11
F3	dorsal attention	limbic	-1.38	3.4e-10
F3	dorsal attention	frontoparietal	-1.16	1.5e-09
F3	dorsal attention	default mode	-1.25	0.0
F4	visual	ventral attention	-1.08	3.2e-07
F4	visual	default mode	-0.74	1.5e-04
F4	somatosensory	ventral attention	-0.99	2.9e-06
F4	somatosensory	default mode	-0.65	1.2e-03
F4	dorsal attention	ventral attention	-1.13	1.5e-07
F4	dorsal attention	limbic	-0.66	4.7e-02
F4	dorsal attention	default mode	-0.79	6.8e-05
F4	ventral attention	frontoparietal	0.73	4.1e-03
thickness	visual	somatosensory	-0.56	1.1e-02
thickness	visual	ventral attention	-1.35	3.9e-13
thickness	visual	limbic	-1.60	1.9e-14
thickness	visual	frontoparietal	-0.67	2.4e-03
thickness	visual	default mode	-1.19	9.7e-14
thickness	somatosensory	ventral attention	-0.79	8.0e-05
thickness	somatosensory	limbic	-1.04	5.0e-06
thickness	somatosensory	default mode	-0.63	4.3e-04
thickness	dorsal attention	ventral attention	-1.21	5.0e-10
thickness	dorsal attention	limbic	-1.45	8.1e-11
thickness	dorsal attention	frontoparietal	-0.52	5.0e-02
thickness	dorsal attention	default mode	-1.05	8.7e-10
thickness	ventral attention	frontoparietal	0.69	2.6e-03
thickness	limbic	frontoparietal	0.93	1.5e-04
thickness	frontoparietal	default mode	-0.53	1.6e-02
myelin	visual	dorsal attention	0.82	8.7e-06
myelin	visual	ventral attention	1.33	0.0
myelin	visual	limbic	1.77	0.0
myelin	visual	frontoparietal	1.20	2.1e-11
myelin	visual	default mode	1.22	0.0
myelin	somatosensory	dorsal attention	0.84	4.3e-06
myelin	somatosensory	ventral attention	1.35	0.0
myelin	somatosensory	limbic	1.79	0.0

myelin	somatosensory	frontoparietal	1.21	7.5e-12
myelin	somatosensory	default mode	1.23	0.0
myelin	dorsal attention	ventral attention	0.51	3.4e-02
myelin	dorsal attention	limbic	0.95	1.9e-05
myelin	limbic	frontoparietal	-0.57	4.8e-02
myelin	limbic	default mode	-0.55	2.8e-02

The significant pairwise differences found for the factors (F1-F4), cortical thickness, and cortical myelination across the Yeo functional networks.
