# Supplementary material for: Mapping the microstructure of human cerebral cortex in vivo with diffusion MRI
Source: Commun Biol. 2025 Jul 22;8:1088. doi: 10.1038/s42003-025-08523-9 (PMC12284103; doi:10.1038/s42003-025-08523-9)
Supplement: Supplementary file 2 — Reporting Summary [file 42003_2025_8523_MOESM2_ESM.pdf]

nature portfolio

Corresponding author(s): Pratik Mukherjee

Last updated by author(s): Jun 3, 2025

Reporting Summary

Nature Portfolio wishes to improve the reproducibility of the work that we publish. This form provides structure for consistency and transparency in reporting. For further information on Nature Portfolio policies, see our Editorial Policies and the Editorial Policy Checklist.

Statistics

For all statistical analyses, confirm that the following items are present in the figure legend, table legend, main text, or Methods section.

n/a	Confirmed	
☐	☒	The exact sample size (*n*) for each experimental group/condition, given as a discrete number and unit of measurement
☐	☒	A statement on whether measurements were taken from distinct samples or whether the same sample was measured repeatedly
☐	☒	The statistical test(s) used AND whether they are one- or two-sided<br>*Only common tests should be described solely by name; describe more complex techniques in the Methods section.*
☒	☐	A description of all covariates tested
☐	☒	A description of any assumptions or corrections, such as tests of normality and adjustment for multiple comparisons
☐	☒	A full description of the statistical parameters including central tendency (e.g. means) or other basic estimates (e.g. regression coefficient) AND variation (e.g. standard deviation) or associated estimates of uncertainty (e.g. confidence intervals)
☐	☒	For null hypothesis testing, the test statistic (e.g. *F*, *t*, *r*) with confidence intervals, effect sizes, degrees of freedom and *P* value noted<br>*Give P values as exact values whenever suitable.*
☒	☐	For Bayesian analysis, information on the choice of priors and Markov chain Monte Carlo settings
☒	☐	For hierarchical and complex designs, identification of the appropriate level for tests and full reporting of outcomes
☐	☒	Estimates of effect sizes (e.g. Cohen's *d*, Pearson's *r*), indicating how they were calculated

Our web collection on statistics for biologists contains articles on many of the points above.

Software and code

Policy information about availability of computer code

Data collection	No software was used for data collection as no novel data were collected as part of this study.
Data analysis	All code used in the data analysis is available on github: https://github.com/ucsfncl/diffusion_neuromaps

For manuscripts utilizing custom algorithms or software that are central to the research but not yet described in published literature, software must be made available to editors and reviewers. We strongly encourage code deposition in a community repository (e.g. GitHub). See the Nature Portfolio guidelines for submitting code & software for further information.

Data

Policy information about availability of data

All manuscripts must include a data availability statement. This statement should provide the following information, where applicable:

- Accession codes, unique identifiers, or web links for publicly available datasets
- A description of any restrictions on data availability
- For clinical datasets or third party data, please ensure that the statement adheres to our policy

The DTI, DKI, NODDI, and MAP-MRI cortical maps can be downloaded at https://github.com/ucsfncl/diffusion_neuromaps. HCP-YA data can be accessed and downloaded at https://www.humanconnectome.org. Volumetric PET images can be found at https://github.com/netneurolab/hansen_receptors or accessed using the neuromaps package: https://github.com/netneurolab/neuromaps. Other relevant cortical maps can also be found using the neuromaps package. BigBrain related data can be found using the BigBrainWarp package: https://github.com/caseypaquola/BigBrainWarp.

Research involving human participants, their data, or biological material

Policy information about studies with human participants or human data. See also policy information about sex, gender (identity/presentation), and sexual orientation and race, ethnicity and racism.

Reporting on sex and gender	Data on sex and gender for the HCP-YA cohort and the MGH-USC dataset is provided by publicly available metadata. We did not perform any gender-based and/or sex-based analysis in this manuscript because most of our inferences are drawn comparing diffusion MRI derived cortical maps to other neurological maps, which are not specific for or corrected for sex and/or gender.
Reporting on race, ethnicity, or other socially relevant groupings	Race, ethnicity, and other socially relevant groupings is provided by publicly available metadata, but we did not perform any analysis based on these metrics in this manuscript because most of our inferences are drawn comparing diffusion MRI derived cortical map to other neurological maps, which are specific for or corrected for race, ethnicity, or socially relevant groupings.
Population characteristics	All participants were from the HCP-Young Adult cohort (N=962, aged 21-35, 46% male) or the MGH-USC dataset (n=35)
Recruitment	No participants were recruited for this study – we only used publicly available data.
Ethics oversight	This study used only publicly available data.

Note that full information on the approval of the study protocol must also be provided in the manuscript.

Field-specific reporting

Please select the one below that is the best fit for your research. If you are not sure, read the appropriate sections before making your selection.

☒ Life sciences ☐ Behavioural & social sciences ☐ Ecological, evolutionary & environmental sciences

For a reference copy of the document with all sections, see nature.com/documents/nr-reporting-summary-flat.pdf

Life sciences study design

All studies must disclose on these points even when the disclosure is negative.

Sample size	Sample sizes were not predetermined and we used all subjects that passed quality control. The sample size (n=962) was sufficient to derive stable and accurate estimates of group averages, intersubject coefficients of variation, and laterality indices of various diffusion MRI related cortical metrics. This is further supported by the low coefficients of variation and high intra-class correlation coefficients that we achieve. Furthermore, all of the statistical testing conducted in this manuscript was conducted region-wise. The sample size of the MGH-USC dataset (n=35) is sufficient to establish reproducibility of group-averaged maps and factors.
Data exclusions	We excluded data only on the basis of quality control due to known anatomical anomalies, segmentation and surface errors, temporal head coil instability, and model fitting irregularities which have been previously reported (see Marcus, D. S. et al. Human Connectome Project informatics: Quality control, database services, and data visualization. Neuroimage 80, 202–219 (2013)).
Replication	Replication of the main findings were tested based on the test-retest portion of the HCP young adult dataset by computing the test-retest coefficient of variation and intra-class correlation coefficient of each of the diffusion MRI derived cortical maps. We were able to derive similar group-averaged maps and factors using the MGH-USC dataset with and without the inclusion of high b-value shells.
Randomization	No data were collected in this study and no experimental groups were constructed in this study.
Blinding	There were no group comparisons in our study, nor was it an interventional study – hence no blinding was necessary.

Reporting for specific materials, systems and methods

We require information from authors about some types of materials, experimental systems and methods used in many studies. Here, indicate whether each material, system or method listed is relevant to your study. If you are not sure if a list item applies to your research, read the appropriate section before selecting a response.

Materials & experimental systems

n/a	Involved in the study
☒	☐ Antibodies
☒	☐ Eukaryotic cell lines
☒	☐ Palaeontology and archaeology
☒	☐ Animals and other organisms
☒	☐ Clinical data
☒	☐ Dual use research of concern
☒	☐ Plants

Methods

n/a	Involved in the study
☒	☐ ChIP-seq
☒	☐ Flow cytometry
☐	☒ MRI-based neuroimaging

Plants

Seed stocks: No seed stocks were used.

Novel plant genotypes: No novel plant genotypes were produced.

Authentication: Since no seed stocks were used and no novel plant genotypes were produced, we did not have any authentication procedures.

Magnetic resonance imaging

Experimental design

Design type: Publicly available structural data (T1w/T2w and diffusion MRI from HCP-YA & MGH-USC) alone were used, hence no design type was applicable.

Design specifications: Only structural and diffusion MRI data was used, so no design specification was necessary.

Behavioral performance measures: No behavioral performance measures were taken.

Acquisition

Imaging type(s): structural, diffusion

Field strength: 3T

Sequence & imaging parameters: We used preprocessed data from the HCP-1200 release of the HCP-YA cohort. This release provides cross sectional 3T images (preprocessed using FreeSurfer version 5.3.0-HCP). Images were 0.7mm isotropic, FOV 224x224 mm, TI=1000ms, TR=2400 ms, flip angle 8 degrees.
We also used data from the MGH-USC dataset (see Fan et al. MGH-USC Human Connectome Project datasets with ultra-high b-value diffusion MRI. Neuroimage. doi: 10.1016/j.neuroimage.2015.08.075). Images were 1mm isotropic, FOV 256 x 256 mm, TI=1100 ms, TR=2530, flip angle 7 degrees

Area of acquisition: whole brain

Diffusion MRI ☒ Used ☐ Not used

Parameters: HCP: Spin echo EPI, TR=5520 ms, TE=89.5 ms, flip angle 78 degrees, FOV 210x180, b-values 1000, 2000, and 3000 s/mm2
MGH-USC: Spin echo EPI, TR=8800 ms, TE=57 ms, FOV 210 x 210 mm, b-values 1000, 3000, 5000, and 10000 s/mm2

Preprocessing

Preprocessing software: We used the preprocessed structural and diffusion MRI data provided from the HCP-YA cohort.

Normalization: Normalization steps were defined by the default recon-all pipeline from FreeSurfer v5.3 used in the HCP-YA preprocessing. These included linear and non-linear registration and intensity normalization.

Normalization template: The fsLR-32k template was used.

Noise and artifact removal: We used Marchenko-Pastur Principal Component Analysis denoising (see Veraart, J. et al. Denoising of diffusion MRI using

Noise and artifact removal	random matrix theory. Neuroimage 142, 394 (2016)) followed by Rician debiasing (see Gudbjartsson, H. & Patz, S. The rician distribution of noisy mri data. Magn Reson Med 34, 910–914 (1995)).
Volume censoring	Volume censoring was not applied

Statistical modeling & inference

Model type and settings	We used multiple linear regression models with dominance analysis as well as partial least squares correlation analysis.
Effect(s) tested	No effects were tested.

Specify type of analysis: ☐ Whole brain ☐ ROI-based ☒ Both

Anatomical location(s)	Locations were based on four predefined parcellations – the Glasser, Desikan Killiany, von-Economo and Koskinas, and Mesulam.
Statistic type for inference (See Eklund et al. 2016)	No voxel-wise or cluster-wise inference was conducted.
Correction	We performed FDR correction to adjust for multiple comparisons.

Models & analysis

n/a	Involved in the study
☒	☐ Functional and/or effective connectivity
☒	☐ Graph analysis
☐	☒ Multivariate modeling or predictive analysis

Multivariate modeling and predictive analysis	We used multiple linear regression models with distance-based cross-validation analysis to determine how predictive our diffusion MRI derived features were and dominance analysis to determine how important each individual feature was to the multivariate prediction. We also perform partial least squares correlation analysis to link our diffusion MRI derived features with neurotransmitter receptor/transporter distribution as well as task-based fMRI activations. Finally, we include a multivariate structural covariance network, which includes all of the diffusion MRI derived measures as well as cortical thickness and cortical myelination.
